# Asiatic acid from *Centella asiatica* alleviates renal fibrosis: coordinated modulation of the gut–kidney axis and retinol metabolism

**DOI:** 10.3389/fnut.2026.1802674

**Published:** 2026-07-10

**Authors:** Liwei Zhu, Ziyun Xu, Xiaoyan Chang, Jingyi Wu, Shutao Chen, Yuanyuan Jiang, Jiayu Guo, Zhenfang Du, Haiyong Chen, Sheng Qiang

**Affiliations:** 1 Department of Nephropathy, Zhangjiagang TCM Hospital Affiliated to Nanjing University of Chinese Medicine, Suzhou, China; 2 Department of Pharmacy, Zhangjiagang TCM Hospital Affiliated to Nanjing University of Chinese Medicine, Suzhou, China; 3 Translational Medical Innovation Center, Zhangjiagang TCM Hospital Affiliated to Nanjing University of Chinese Medicine, Suzhou, China; 4 School of Chinese Medicine, The University of Hong Kong, Hong Kong SAR, China

**Keywords:** asiatic acid, chronic kidney disease, gut microbiota, metabolomics, renal fibrosis, retinol metabolism

## Abstract

**Introduction:**

Chronic kidney disease (CKD) is a progressive condition characterized by renal fibrosis, with limited therapeutic options. Asiatic acid (AA), a triterpenoid from *Centella asiatica*, has shown renoprotective potential, but its mechanisms remain unclear.

**Methods:**

An adenine-induced CKD mouse model was established in C57BL/6 mice. AA was administered orally at 10 or 30 mg/kg for 8 weeks. Antibiotic depletion experiments were performed to assess gut microbiota dependence. Renal function, histopathology, and fibrosis markers were evaluated. Multi-omics analyses integrated 16S rRNA sequencing, renal untargeted metabolomics, transcriptomics, and MetOrigin-based tracing. *In vitro*, human proximal tubular HK-2 cells were stimulated with TGF-β1 to assess the effects of key metabolites on fibrotic injury and oxidative stress.

**Results:**

AA significantly improved renal function, reduced tubular injury and collagen deposition, and restored gut barrier integrity. Antibiotic depletion experiments demonstrated that an intact gut microbiota is required for the renoprotective effects of AA supporting a functional link along the gut-kidney axis. AA remodeled the gut microbiota, notably enriching *Lachnospiraceae NK4A136 group*. Renal metabolomics and transcriptomics integration identified retinol metabolism as the key renal pathway rescued by AA, with 9-cis-retinal and 4-hydroxyretinoic acid negatively correlated with injury markers. *In vitro*, 9-cis-retinal suppressed TGF-β1-induced fibrotic markers and oxidative stress.

**Discussion:**

AA alleviates CKD in association with remodeling of the gut microbiota and reprogramming of renal retinol metabolism, supporting its potential as a dietary supplement for countering renal fibrosis and supporting kidney health.

## 1 Introduction

Chronic kidney disease (CKD) affects over 10% of the global population (1) and is estimated to become the fifth leading cause of death worldwide by 2040 (2). Current treatment approaches involve managing symptoms and slowing disease progression with medications and dietary strategies such as protein restriction and sodium reduction. In the later stages, both primary and secondary CKD causes display consistent histopathological evidence of tubulointerstitial and glomerular fibrosis. Renal fibrosis (RF) is a key pathogenic mechanism that leads to the progressive loss of nephrons, functional decline, and eventually the development of ESRD (3).

Emerging evidence has identified specific metabolites that may exacerbate kidney injury and fibrosis by activating pro-fibrotic pathways, thereby presenting potential therapeutic targets for CKD treatment (4). Disruptions in amino acid, lipid, vitamin, and carbon metabolism exacerbate oxidative stress, inflammation, and fibrosis, further impairing renal function (5, 6). Metabolites are synthesized by both the host organism and the gut microbiota (7). Dysbiosis of the gut microbiota, impaired intestinal barrier function, and altered host metabolism collectively contribute to renal inflammation, oxidative stress, and fibrosis. Identifying gut microbial metabolites associated with RF and investigating the effects of the gut microbiota and its metabolites on renal metabolism are essential for a comprehensive understanding of CKD pathogenesis (8). Natural bioactive compounds derived from edible plants, such as flavonoids and terpenoids, have shown potential in restoring microbial homeostasis and improving metabolic health, offering a complementary approach to conventional RF management (9–11).

*Centella asiatica* (L.) Urb., commonly known as “ji xue cao” in China, or “Gotu Kola” in other regions, has a long history of culinary and medicinal use (12). Its extracts are widely used as dietary supplements around the world, and its leaves are consumed as a vegetable or brewed as a tea (13). Asiatic acid (AA) is a triterpenoid compound extracted from *Centella asiatica* and is regarded as the major active ingredient (14). Previous research has demonstrated that AA has anti-inflammatory, antioxidant (15), and anti-fibrotic properties (16). However, the therapeutic potential of this compound is limited due to its low solubility and oral bioavailability (17). It is therefore hypothesized that the gut microbiota may represent a crucial pathway in the mechanism of action of AA. Nonetheless, the role of host-microbiota interactions and gut microbiota co-metabolism in renal fibrosis has yet to be thoroughly investigated.

In this study, we utilized an integrated multi-omics approach, incorporating 16S rRNA sequencing, untargeted metabolomics, transcriptomics, and host–microbiota co-metabolism analysis, to elucidate the renoprotective mechanisms of AA in a mouse model of adenine-induced renal fibrosis. Antibiotic (ABX) depletion experiments indicate that the gut microbiota is required for the renoprotective effects of AA, supporting a functional link along the gut–kidney axis. Our findings suggest that AA alleviates CKD in association with remodeling of the gut microbiota and reprogramming of renal retinol metabolism. The results of our study offer novel insights into the potential of AA as a microbiota-targeting dietary agent for CKD management, thereby underscoring the therapeutic role of natural triterpenoids in the gut–kidney axis.

## 2 Materials and methods

### 2.1 Drugs and reagents

AA was procured from MedChemExpress (HY-N0194, USA). A mouse diet enriched with 0.25% adenine was obtained from Jiangsu Xietong Pharmaceutical Bio-Engineering Co., Ltd. (Nanjing, China) (XT19031).

### 2.2 Animals

Eight-week-old male C57BL/6 mice (20 ± 2 g) were acquired from Skobes Biotechnology Co., Ltd. (Anyang, China) (Laboratory Animal Production License: SCXK-2020-0005) and kept under controlled conditions (25 ± 2 °C, 65% ± 5% humidity, 12-h light/dark cycle) with free access to food and water. After 7 days of acclimatization, mice were randomly assigned to four groups (*n* = 6 per group): Control (C, normal diet), CKD model (M, 0.25% adenine diet), Low-dose AA (AL, adenine diet + 10 mg/kg AA), High-dose AA (AH, adenine diet + 30 mg/kg AA). The doses of AA used in this study (10 and 30 mg/kg/day) are within the range commonly used in rodent models of kidney disease. Human equivalent doses based on body surface area (18) would be approximately 0.81 and 2.43 mg/kg/day. AA was initially dissolved in DMSO as a stock solution, which was then diluted with saline to achieve a working solution containing 1% DMSO. The working solution was then subjected to ultrasonic agitation to ensure its dissolution into a homogeneous suspension, thus creating a backup solution. The control and model groups received an equivalent volume of vehicle (1% DMSO in saline) by oral gavage daily, following the same administration protocol of AA groups. The CKD model was established by administering adenine-enriched chow for 4 weeks, as previously described (19, 20). Concurrently, AL and AH groups received daily oral gavage of AA at respective doses, while the control animals were maintained on adenine-free chow. Weeks 5–8, all groups were transitioned to standard diet while AA treatment continued.

For antibiotic (ABX) depletion experiment, mice were orally gavaged daily for 1 week with a broad-spectrum antibiotic cocktail (ampicillin 100 mg/kg, vancomycin 50 mg/kg, neomycin 100 mg/kg, metronidazole 100 mg/kg) to deplete the gut microbiota, followed by adenine-induced CKD modeling and AA treatment (30 mg/kg).

Urine samples were collected at baseline, week 4, and week 8 using metabolic cages, then centrifuged at 3,000 rpm for 10 min at 4 °C, and preserved for analysis.

After the 8-week intervention, mice were deeply anesthetized by inhalation of 4% isoflurane in oxygen (flow rate 1 L/min). Blood was then drawn via cardiac puncture, which resulted in rapid exsanguination and death, serving as the method of euthanasia. One kidney was fixed in paraformaldehyde for histopathological processing, the other was frozen in liquid nitrogen for further analyses. Colon tissues were collected for subsequent molecular biology study. The investigators performing outcome assessments were blinded to group allocation.

### 2.3 Serum and urine biochemical analysis

Renal function parameters were evaluated utilizing commercial assay kits for the quantification of serum creatinine (SCR, E-BC-K188-M, Elabscience, China) and blood urea nitrogen (BUN, E-BC-K183-M, Elabscience, China). The urinary albumin-to-creatinine rate (UACR) was measured using the creatinine colorimetric assay kit and microalbuminuria ELISA kit (E-EL-M0792, Elabscience, China). Urinary levels of kidney injury molecule-1 (Kim-1) were quantified via an ELISA assay (E-EL-M3039, Elabscience, China). All procedures were conducted in accordance with the manufacturer's protocols.

### 2.4 Histopathological analysis

Paraffin-embedded kidney tissues were sectioned into slices of 4 μm thickness. The sections underwent dewaxing in xylene, followed by rehydration through a gradient ethanol series, and were subsequently stained using a hematoxylin-eosin (H&E) staining kit (G1076, Servicebio, China), Masson's trichrome kit (G1006, Servicebio, China), or the modified Sirius Red staining kit (G1078, Servicebio, China). The sections with the Sirius Red staining were imaged under a polarized light microscope to visualize collagen fibers.

### 2.5 Immunofluorescence

After dewaxing, antigen repair, and blocking, colon tissue sections were incubated overnight at 4 °C with primary antibodies, then treated with a secondary antibody. The primary antibodies utilized included ZO-1 (1:200, 21773-1-AP, Proteintech, China), Occludin (1:200, sc-133256, Santa Cruz, USA), E-cadherin (1:200, GB11082, Servicebio, China). The secondary antibodies comprised Cy3 conjugated Goat Anti-Rabbit IgG (1:300, GB21303, Servicebio, China), or Alexa Fluor 488-conjugated Goat Anti-Rabbit IgG (1:400, GB25303, Servicebio, China). Subsequently, the nuclei were stained with 4′,6-diamidino-2-phenylindole (DAPI, G1012, Servicebio, China). Observations and image capture were conducted using fluorescence microscopy (Nikon Eclipse C1, Tokyo, Japan).

### 2.6 Western blots

Mouse kidney tissue was lysed using RIPA lysis buffer (P0013C, Beyotime, China), protein concentrations were determined employing a BCA protein quantification kit. Equivalent protein quantities were resolved via SDS-PAGE and subsequently transferred onto PVDF membranes. Following blocking with a protein-free blocking buffer (P0252, Beyotime, China), PVDF membranes were incubated with primary antibodies at 4 °C overnight. The membranes were then incubated with HRP-conjugated secondary antibodies for 1 h at room temperature. Protein bands were visualized with ECL kits and quantified with ImageJ software. Antibodies used in the study included α-SMA (1:1,000, #19245, CST, USA), TGF-β1 (1:1,000, ab215715, Abcam, UK), COL1 (1:1,000, #72026, CST, USA), ALDH1A1 (1:1,000, 15910-1-AP, Proteintech, China), CYP2A6 (1:1,000, 21721-1-AP, Proteintech, China), RARA (1:1,000, 10331-1-AP, Proteintech, China), and β-actin (1:10,000, HRP-66009, Proteintech, China). β-actin served as the internal loading control.

### 2.7 16S rRNA gene sequencing analysis

Fecal samples from mice were aseptically collected in sterile tubes and stored at −80 °C. Genomic DNA was extracted using the CTAB method, followed by purification with magnetic beads. The V4 region of 16S rRNA was PCR-amplified with barcoded primers (515F/806R) using Phusion^®^ High-Fidelity PCR Master Mix. PCR products were gel-purified, and libraries were quantified via Qubit^®^ and Q-PCR. Sequencing was done on the Illumina NovaSeq6000 platform. Raw reads were merged with FLASH (v1.2.7) and filtered with fastp (v0.23.1) to produce clean tags. Chimeras were removed using vsearch (v2.16.0) against the SILVA138 database. Operational taxonomic units (OTUs) were clustered at 97% similarity with Uparse (v7.0.1) and annotated with Mothur. Alpha and beta diversity indices were calculated. Linear discriminant analysis (LDA) effect size (LEfSe) identified differentially abundant taxa. Species co-occurrence networks were built using Pearson correlation. Two-way orthogonal partial least Squares (O2PLS) analysis integrated multi-omics data to identify key variables. Spearman correlation heatmaps between metabolites and microbial genera were generated using bioinformatics tools (https://www.bioinformatics.com.cn) (21). Bioinformatics processing was conducted on the Novogene Cloud Platform (https://magic.novogene.com/).

### 2.8 Kidney untargeted metabolomics analysis

Kidney untargeted metabolomics were conducted using a Vanquish UHPLC system and an Orbitrap Q ExactiveTMHF-X mass spectrometer (Thermo Fisher, Germany) at Novogene Co., Ltd. in Beijing. Following sample preparation, chromatography, and mass spectrometry, metabolic profiling and data processing were conducted. QC samples ensured system repeatability and stability. Potential differential metabolites were identified using PCA and PLS-DA via metaX (1.4.2). Volcano plots, incorporating VIP scores, log2(FC), and –log10(*p*-value), were created with ggplot2 in R (4.0.0). Cluster heatmaps were generated using Pheatmap (1.0.11) in R, with metabolite data normalized using z-score transformation. KEGG pathway and SMPDB pathway enrichment analysis were performed using MetaboAnalyst 6.0. Additionally, MetOrigin (https://metorigin.met-bioinformatics.cn) was used to perform traceability analysis on differential metabolites (7).

### 2.9 RNA sequencing

Total RNA underwent quality control using the RNA Nano 6000 Assay Kit of the Bioanalyzer 2100 system (Agilent Technologies, CA, USA). Sequencing libraries were constructed from qualified RNA. Briefly, mRNA was enriched via poly-A selection, fragmented, and reverse-transcribed into double-stranded cDNA. After end repair and adapter ligation, fragments of the desired size were selected via bead-based purification and amplified by PCR. The final libraries were quantified and assessed for quality before being sequenced on an Illumina NovaSeq platform to generate 150 bp paired-end reads. For data analysis, raw reads were processed to remove adapters and low-quality sequences. The clean reads were then aligned to a reference genome using HISAT2 v2.0.5. Gene expression levels were quantified as FPKM using featureCounts v1.5.0-p3. Differential expression analysis between groups was performed using DESeq2 R package (1.20.0), with genes possessing an adjusted FDR (adjusted *p*-value) ≤ 0.05 and |log2(Fold Change)| ≥1 considered significant. Finally, KEGG pathway enrichment analysis was conducted on the differentially expressed genes (DEGs) to identify affected biological pathways.

### 2.10 Cell culture and treatment

Human proximal tubular epithelial cells (HK-2) were obtained from Procell Life Science & Technology Co., Ltd. (Wuhan, China). The cells were maintained in a differentiated state using DMEM/F12 medium (Gibco, USA) with 1% penicillin-streptomycin (C0222, Beyotime, China) and 10% fetal bovine serum (FBS, Biological Industries, Israel). The cells were cultured in a humidified incubator set to 37 °C with a 5% CO_2_ atmosphere. A model of fibrotic cell injury was established using transforming growth factor-β1 (TGF-β1, 100-21, PeproTech, USA) by 10 ng/ml for 24h. 9-cis-retinal (HY-W009310, MCE, USA) at 10 μM, 4-hydroxyretinoic acid (HY-125904, MCE, USA) at 90 nM, AA at 15 μg/ml and Methoxsalen (HY-30151, MCE, USA) at 50 μM were used in HK-2 following a screening process to determine the optimal concentration.

### 2.11 Detection of reactive oxygen species production and mitochondrial membrane potential

For detection of cytosolic reactive oxygen species (ROS) and mitochondrial membrane potential (MMP), 5 × 10^3^ cells were seeded in each well (a 96-well plate) 1 day before the experiment. Following treatment with TGF-β1 /and 4-hydroxyretinoic acid, ROS and MMP were detected separately with the ROS assay kit (S0035S, Beyotime, China) and the MT-1 MitoMP detection kit (MT13, Dojindo, Japan), following the manufacturer's instructions. The results were detected using a fluorescence microplate reader and a fluorescence microscope, respectively.

### 2.12 Statistical analysis

A *post-hoc* power analysis (G^*^Power 3.1) based on a two-tailed *t*-test, an α of 0.05, *n* = 6 per group, and the observed effect size for serum creatinine (Cohen's *d* = 2.3) (22) yielded a power of 0.95, indicating that the sample size was sufficient to reliably detect the renoprotective effects of AA. All continuous data were presented as mean ± standard error of the mean (SEM) from at least three independent experiments. Following normality testing, statistical analysis was performed using a two-tailed Student's *t*-test to analyze two groups and one-way analysis of variance (ANOVA) with Tukey's test to analyze more than two groups with GraphPad Prism software (v.9.3.1, USA). For non-normal distribution, Kruskal–Wallis test followed by Dunn's test was used for multiple comparisons. A *P*-value of less than 0.05 was considered indicative of statistical significance.

## 3 Results

### 3.1 AA ameliorates renal dysfunction in CKD mice

**Figure 1:**
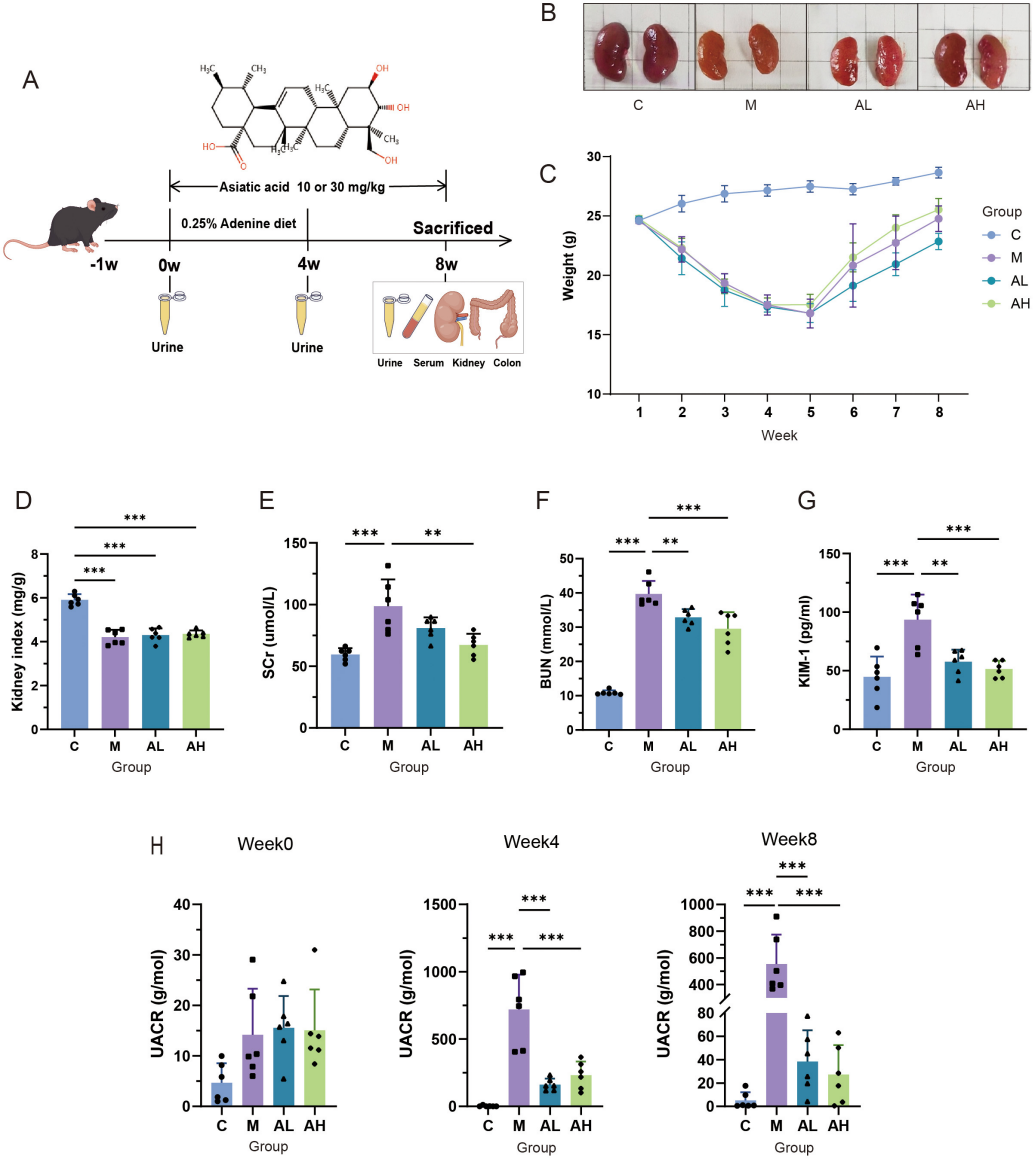
The therapeutic effects of AA on CKD mice. **(A)** Diagram of the animal experimental design. **(B)** Photographs of the kidneys from each group, with a scale of 1 grid = 0.5 cm. **(C)** Body weight changes of mice in each group throughout the experimental period. **(D)** Kidney coefficient of mice in each group, calculated as kidney weight/body weight. **(E)** Scr levels in mice from each group (*n* = 6). **(F)** BUN levels in mice from each group (*n* = 6). **(G)** Urinary KIM-1 levels in mice from each group (*n* = 6). **(H)** UACR levels of mice from each group at weeks 0, 4, and 8 (*n* = 6). C = control, M = CKD/adenine, AL = low dose AA, AH = high dose AA. After normality testing, one-way ANOVA followed by Tukey's test or Kruskal–Wallis test followed by Dunn's test was used for multiple comparisons. *P < 0.05, **P < 0.01, ***P < 0.001.

As shown in Figure 1, CKD mice fed with adenine developed remarkable renal atrophy, pale coloration and granular surfaces, and lower body weight compared to normal diet mice, whereas AA treatments partially restored these parameters in CKD mice. Consistently, AA treatment increased kidney function and attenuated kidney injuries in CKD mice as demonstrated by the reduced levels of Scr, BUN, and Kim-1 in AA treatment groups (Figures 1E–G). At baseline, the UACR levels in all groups were comparable. However, by 4 weeks, the model group showed a significant increase, whereas the AL and AH groups experienced a marked decrease compared to the model group (*P* < 0.001; Figure 1H). Collectively, these results indicate that AA can prevent adenine-induced renal damage.

### 3.2 AA ameliorates renal histopathological damage and fibrosis in CKD mice

**Figure 2:**
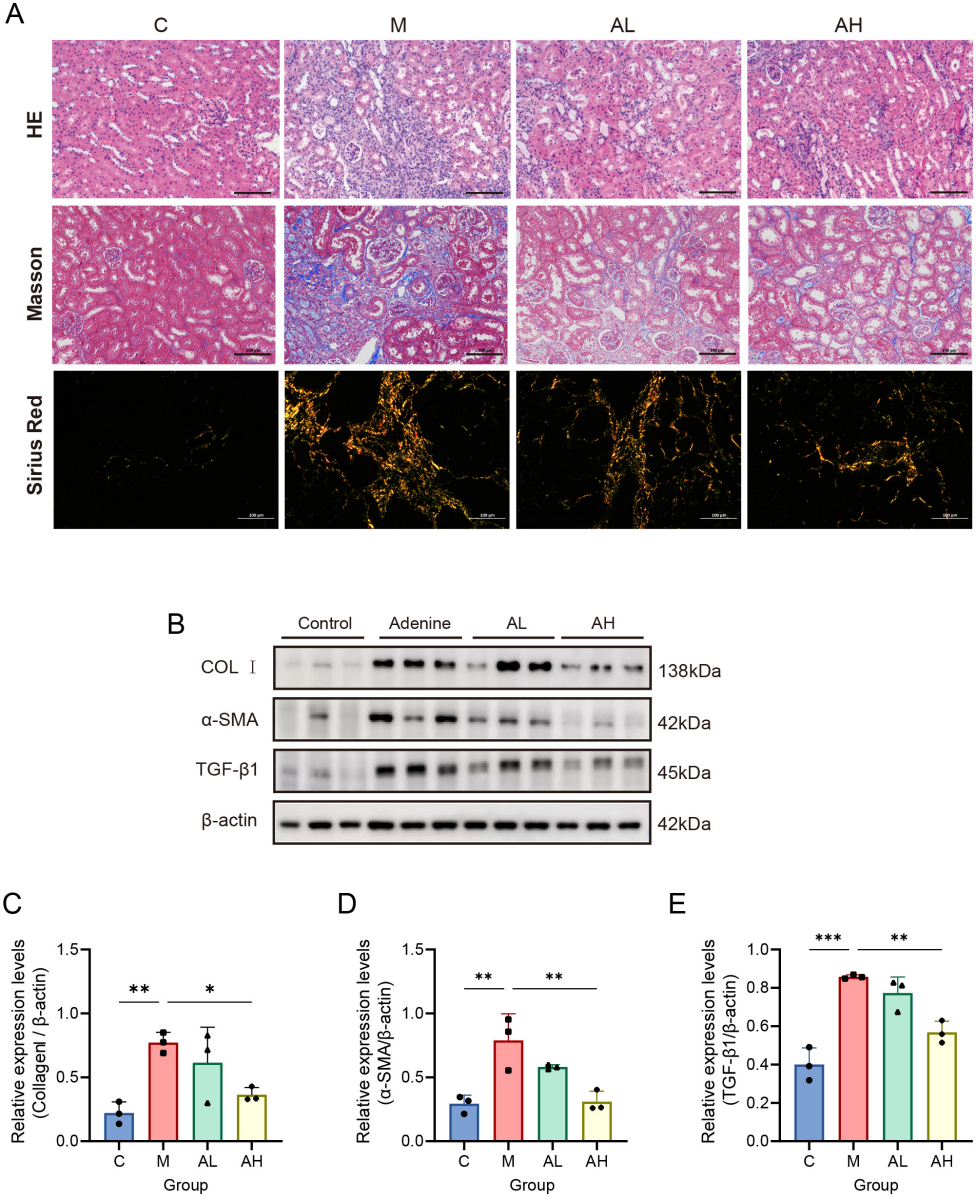
AA ameliorate kidney pathological changes and fibrosis in CKD mice. **(A)** H&E, Masson's trichrome, and Sirius Red staining (under polarized light) for each group ( × 200). **(B)** Western blotting results for kidney Col-I, α-SMA, and TGFβ1 in mice from each group (*n* = 3). **(C–E)** Semi-quantitative analysis of each protein. After normality testing, one-way ANOVA followed by Tukey's test or Kruskal–Wallis test followed by Dunn's test was used for multiple comparisons. *P < 0.05, **P < 0.01, *n* = 3.

H&E staining showed that CKD mice had notable tubular atrophy, dilatation, vacuolization, and epithelial cell flattening and shedding in their kidneys. Masson's trichrome staining demonstrated substantial collagen deposition in these kidneys. Additionally, polarized Sirius Red staining showed a notable increase in bright red and yellow regions, representing type I collagen, as well as light green regions indicating type III collagen in kidneys of CKD mice (Figure 2A). Treatment with AA alleviated tubular injury and decreased collagen deposition in kidneys of CKD mice. Western blot analysis revealed elevated levels of fibrotic markers, Col-I, α-SMA, and TGFβ1, in the CKD group compared to the control group (*P* < 0.05) while AA treatments decreased these fibrotic markers in kidneys of CKD mice, particularly in the high-dose group (*P* < 0.05; Figures 2B–E). Due to the superior efficacy of high-dose AA, subsequent experiments were performed on the C, M, and AH groups only.

### 3.3 Gut microbiota is required for the renoprotective effects of AA

**Figure 3:**
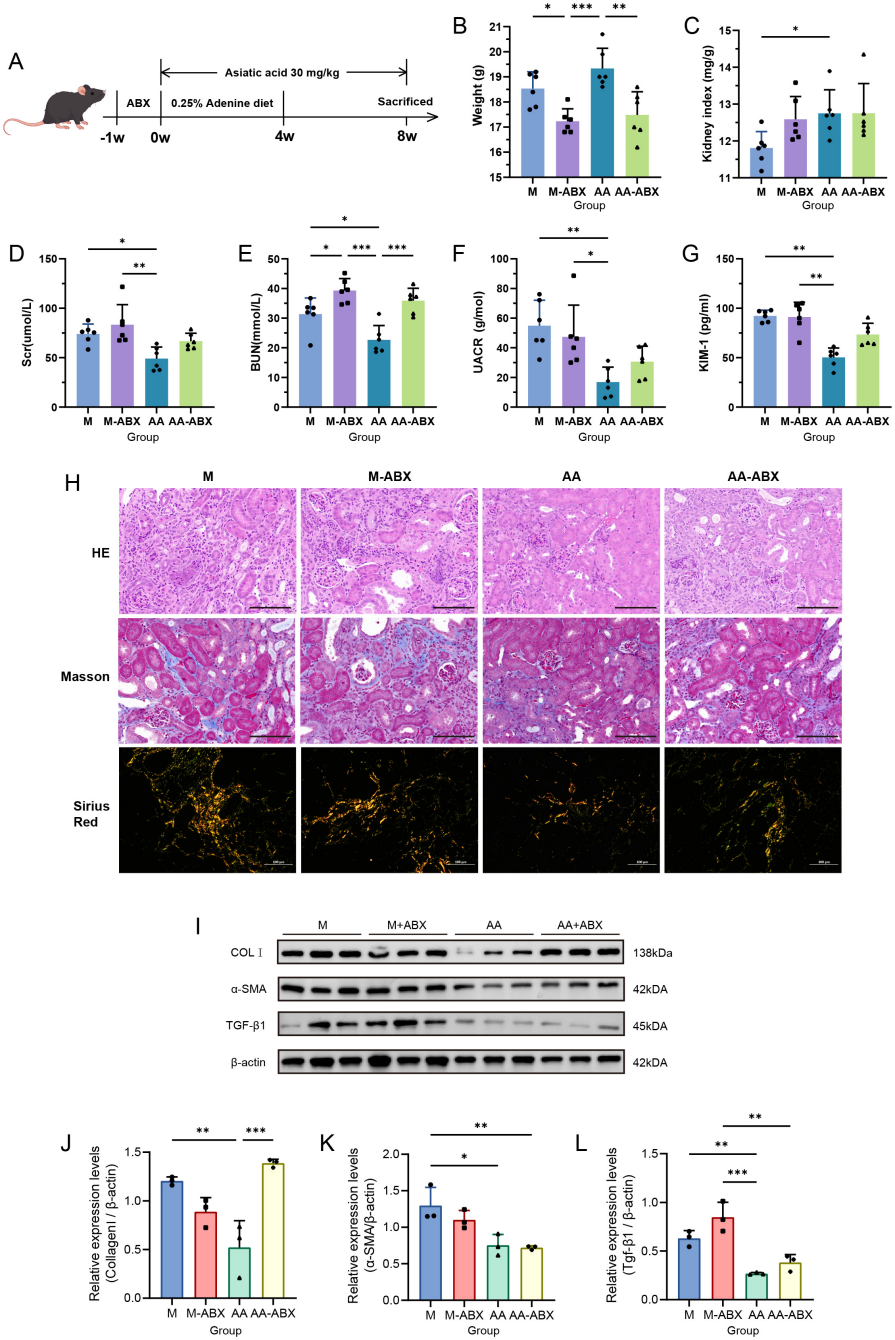
Intact gut microbiota is required for the renoprotective effects of AA in CKD mice. **(A)** Schematic of the experimental timeline. Physiological and renal function parameters across groups: **(B)** body weight, **(C)** kidney index, **(D)** Scr, **(E)** BUN, **(F)** UACR, and **(G)** KIM-1 (*n* = 6). **(H)** Representative images of renal histopathology: H&E staining, Masson's trichrome staining, and Sirius Red staining under polarized light (scale bars = 100 μm). Western blot analysis **(I)** and semi-quantification **(J–L)** of renal fibrotic markers: Collagen I (Col I), α-SMA, and TGF-β (*n* = 3). C = control, M = CKD/adenine, AA = high dose AA. After normality testing, one-way ANOVA followed by Tukey's test or Kruskal–Wallis test followed by Dunn's test was used for multiple comparisons. *P < 0.05, **P < 0.01, ***P < 0.001.

To determine whether the gut microbiota is necessary for the renoprotective actions of AA, we performed an antibiotic depletion experiment. The experimental protocol is illustrated in Figure 3A. ABX treatment resulted in significant body weight loss (Figure 3B) but had minimal impact on the kidney index (Figure 3C). Notably, ABX largely abolished the AA (30 mg/kg)-mediated reduction in BUN (Figure 3E). Although Scr, UACR, and KIM-1 levels tended to decrease in the AA-ABX group, these changes did not reach statistical significance compared with the model group (Figures 3D, F, G). Consistently, the improvements in renal histopathology and fibrosis conferred by AA were attenuated in ABX-treated mice (Figure 3H). COL1 expression was significantly elevated in the AA-ABX group relative to the AA-alone group, whereas α-SMA and TGF-β1 levels remained comparable or showed slight increases (Figures 3I–L). Together, these results indicate that an intact gut microbiota is required, at least in part, for the renoprotective effects of AA.

### 3.4 AA reversal shifts gut microbial composition in CKD mice

**Figure 4:**
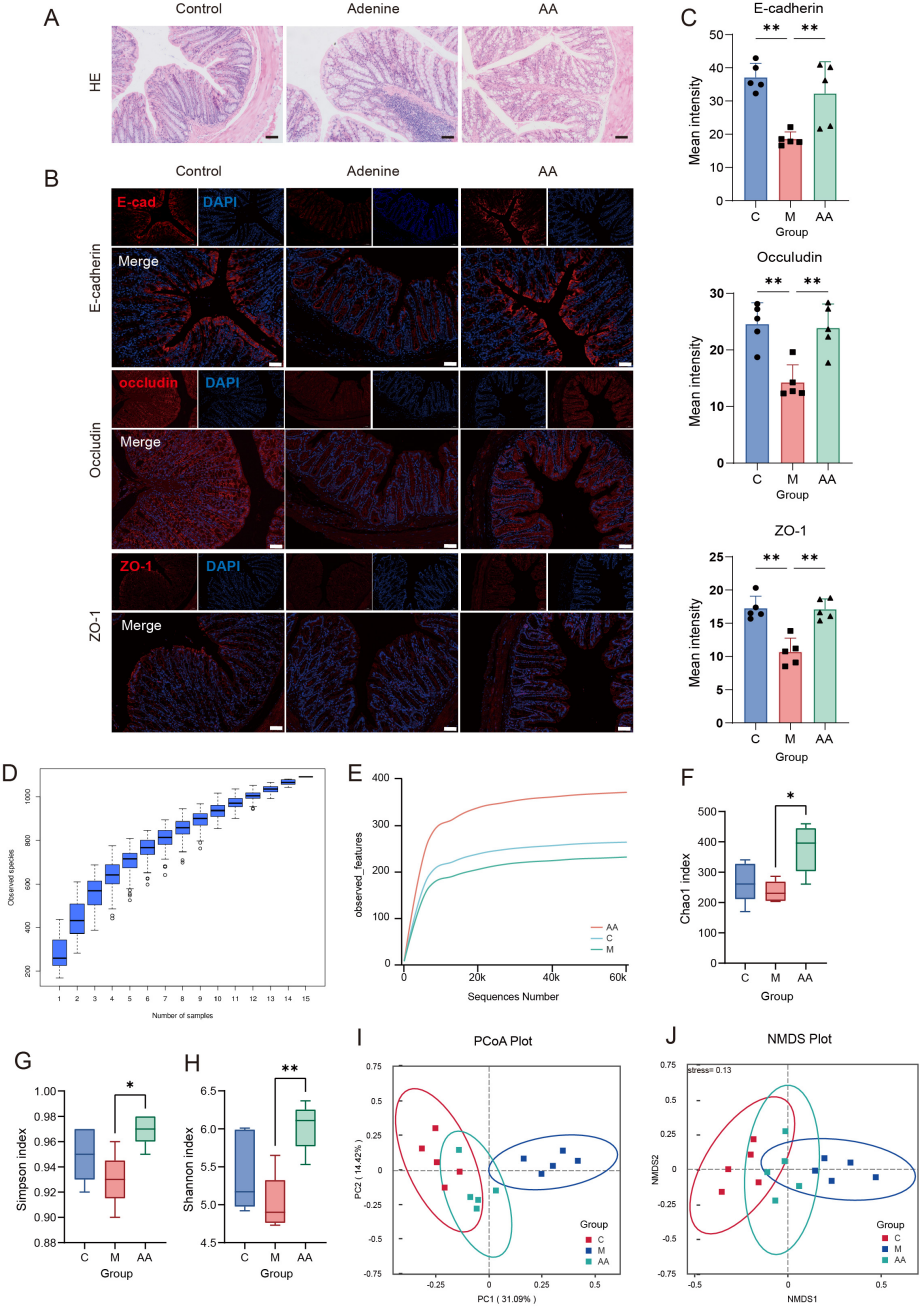
Effects of AA on the gut barrier and the structure of intestinal flora. **(A)** HE staining of colonic tissues across three groups (scale bar: 50 μm). **(B)** Immunofluorescence co-localization of tight junction proteins (E-cadherin, Occludin and ZO-1) in colonic tissues (scale bar: 50 μm). **(C)** Quantitative analysis of mean fluorescence intensity for colonic tight junction proteins. **(D)** Species Accumulation curve for all samples. **(E)** Rarefaction curve among C, M, and AA groups. Boxplots show differences among groups based on **(F)** the Chao1 index, **(G)** Simpson index, and **(H)** Shannon index. **(I)** PCoA plot. **(J)** NMDS plot. AA, high dose AA at 30 mg/kg. *P < 0.05, **P < 0.01, *n* = 5.

To examine colonic pathology and barrier integrity, we studied AA's impact on the colon. H&E staining showed disrupted crypt structures, fewer goblet cells, and weakened epithelial barriers in both adenine and AA groups (Figure 4A). Immunofluorescence revealed disorganized E-cadherin, Occludin, and ZO-1 localization, with lower fluorescence intensity (Figures 4B, C), indicating impaired tight junctions.

16rRNA gene sequencing was used to evaluate gut microbiota structural changes in the C, M, and AA groups. The species accumulation curve plateaued with more samples, indicating sufficient sampling and stable species numbers in the environment (Figure 4D). Similarly, the rarefaction curve leveled off with increased sample reads, suggesting OTU saturation and adequate sequencing depth to reflect community abundance (Figure 4E). Alpha diversity analysis revealed no significant differences between the C and M groups in Chao1, Simpson, and Shannon indexes. However, post-AA intervention, significant differences emerged compared to the M group, indicating increased species abundance, evenness, and diversity in the gut microbiota of mice (Figures 4F–H). Bray-Curtis distances were utilized to conduct principal coordinates analysis (PCoA) and non-metric dimensional scaling (NMDS), both showing clear distinctions among the C, M, and AA groups, suggesting that adenine treatment altered the gut microbiome (Figure 4I). The stress value for the NMDS analysis was less than 0.2, confirming that the analysis accurately reflected the differences between the samples (Figure 4J). These results indicate that AA can effectively modulate the structure of the gut microbiota.

### 3.5 AA alleviates adenine-induced gut microbiota disorders

**Figure 5:**
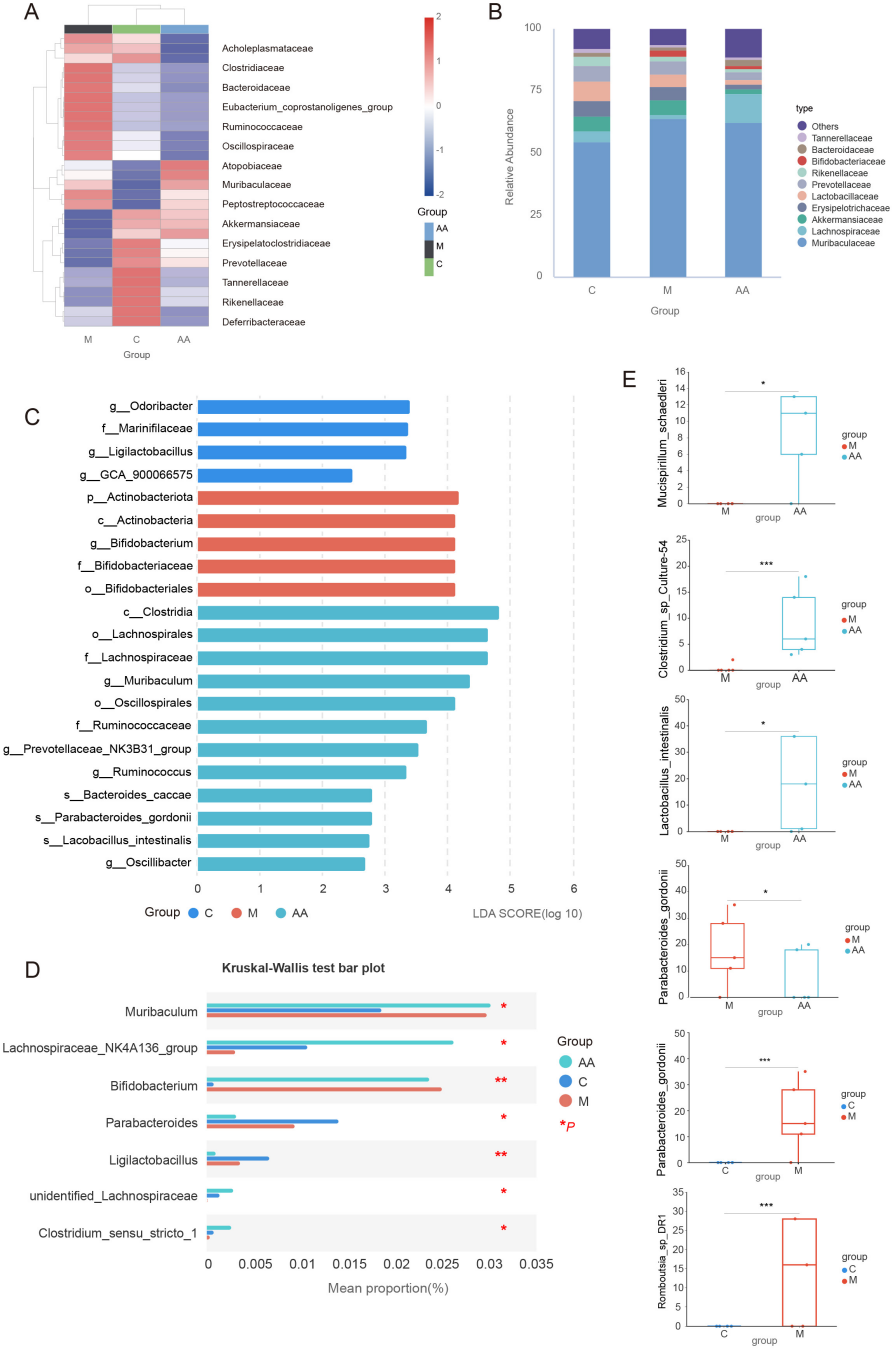
Screening of AA-associated gut microbial biomarkers. **(A)** Heatmap of gut microbiota at the family level among the C, M, and AA groups. **(B)** Taxonomic composition and relative abundance of bacterial communities at the family level among the three groups. **(C)** LEfSe bar plot illustrating the distribution of LDA values (LDA > 2.5, *P* < 0.05) for significantly different taxa across groups. **(D)** Differential genera among the C, M and AA groups, analyzed by Kruskal–Wallis test at the genus level. **(E)** MetagenomeSeq-based analysis of species-level biomarkers. AA, high dose AA at 30 mg/kg. *P < 0.05, **P < 0.01, ***P < 0.001.

To identify specific gut microbiota influenced by AA, we further investigated structural alterations in the microbiota following AA intervention. Family-level hierarchical clustering analysis (HCA) showed that the AA and C groups were more similar in microbial community structure than the AA and M groups (Figure 5A). Based on taxonomic classification, the dominant bacterial families across groups included *Muribaculaceae, Lachnospiraceae, Akkermansiaceae*, and *Erysipelotrichaceae* (Figure 5B). LEfSe analysis revealed dominant bacterial taxa across groups, with microbial biomarkers displayed in a cladogram and a LDA value distribution histogram (LDA > 2.5, *P* < 0.05). In the C group, the genera *Ligilactobacillus, Odoribacter*, and *GCA_900066575* (uncultured genus), along with the family *Marinifilaceae*, were predominant. The M group was characterized by enrichment of the phylum *Actinobacteriota*, class *Actinobacteria*, order *Bifidobacteriales*, family *Bifidobacteriaceae*, and genus *Bifidobacterium*. In contrast, the AA group exhibited enrichment of the class *Clostridia*, orders *Lachnospirales* and *Oscillospirales*, families *Lachnospiraceae* and *Ruminococcaceae*, as well as the genera *Muribaculum* and *Prevotellaceae NK3B31 group* (Figure 5C and Supplementary Figure S1). Kruskal–Wallis test analysis of intergroup differences at the genus level identified *Muribaculum, Lachnospiraceae NK4A136 group, Bifidobacterium* as significantly altered genera (*P* < 0.05), with *Lachnospiraceae NK4A136 group* showing the highest fold increase in the AA group compared to the M group (Figure 5D). At the species level, MetagenomeSeq analysis identified key taxa that were significantly altered by adenine and AA. Compared to the C group, the abundances of *Parabacteroides gordonii* and *Romboutsia sp. DR1* were significantly elevated in the M group. However, AA intervention effectively suppressed the enrichment of *Parabacteroides gordonii*. Furthermore, AA treatment led to the significant enrichment of beneficial species, including *Lactobacillus intestinalis, Clostridium sp. Culture-54*, and *Mucispirillum schaedleri*, compared to the M group (Figure 5E). These results suggest that AA not only promotes probiotics but also counteracts the expansion of specific taxa associated with CKD progression.

### 3.6 AA alleviates kidney metabolic dysregulation in adenine-induced CKD mice

**Figure 6:**
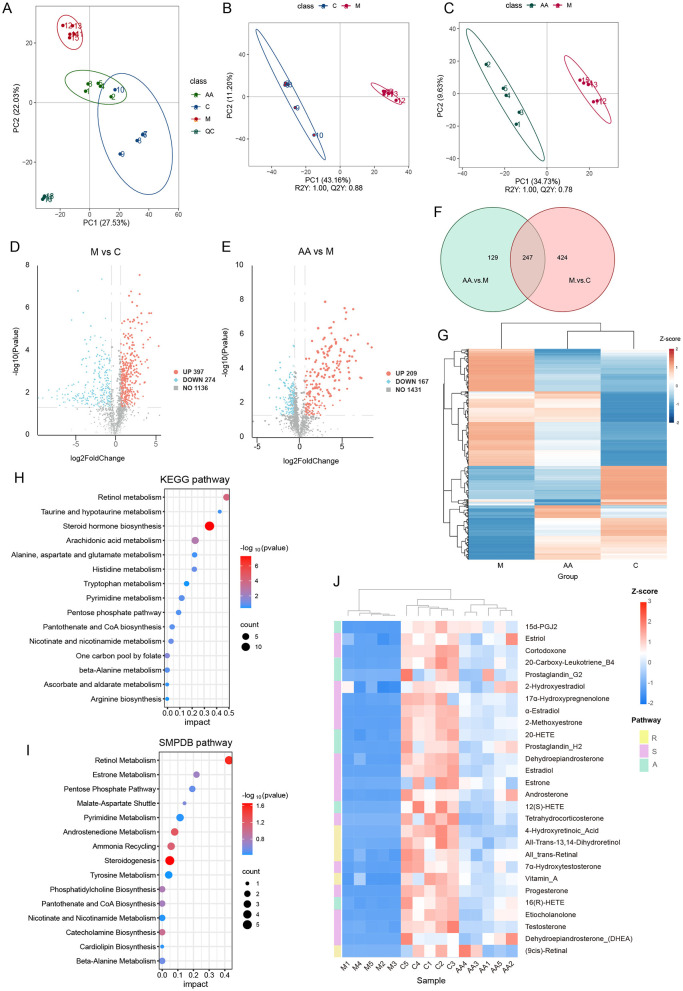
Effect of AA on kidney metabolism in adenine-induced CKD mice. **(A)** PCA score plots among QC, C, M, and AA groups in mixed ion mode. PLS-DA score plots for M vs. C **(B)** and AA vs. M **(C)**. The circles denote the 95% confidence intervals. **(D, E)** Volcano plots of differential metabolites for group M vs. C and group AA vs. M in mixed ion modes. Red and blue-marked metabolites meet the criteria of *P* < 0.05, |log2FoldChange| ≥ 0.58, VIP ≥ 1. **(F)** Venn diagram of differential metabolites for group M vs. C and group AA vs. M. The circles indicate the number of metabolites unique to each group. **(G)** Clustering heatmaps of the differential metabolites among C, M, and AA groups. Horizontal axis represents metabolite clustering, and vertical axis represents sample type; shorter clustering branches indicate higher similarity. **(H)** KEGG enrichment analysis and **(I)** SMPDB enrichment analysis of shared differential metabolites between each pairwise comparison. **(J)** Heatmap of *z*-score normalized metabolite peak intensities across experimental groups. AA, high dose AA at 30 mg/kg. R, retinol metabolism. S, steroid hormone biosynthesis. A, arachidonic acid metabolism.

To examine the impact of AA on kidneys damaged by adenine, we conducted a non-targeted metabolomics analysis on renal tissues. Due to the positive effects of high-dose AA on kidney injuries, we focused on three groups: C, M, and AH. In PCA analysis, QC samples clustered tightly, showing method stability. The M group was distinct from AA and C groups, with AA partially overlapping C (Figure 6A). PLS-DA scores highlighted group differences, with R^2^Y indicating model explanation and Q^2^Y assessing prediction. An R^2^Y higher than Q^2^Y signals a strong model, and values near 1 indicate stability. Both models were robust (Figures 6B, C). The M group showed 397 upregulated and 274 downregulated metabolites compared to C under mixed ion modes (Figure 6D). Conversely, compared to the M group, the AA group displayed 209 significantly upregulated metabolites and 167 significantly downregulated metabolites (Figure 6E). A total of 247 distinct differential metabolites were shared between the two comparative groups (Figure 6F). HCA showed that the C and AA groups were more similar, with shorter branch lengths (Figure 6G). Shared metabolites from these groups underwent pathway analysis using MetaboAnalyst 6.0. The top five pathways from KEGG analysis were Retinol metabolism, Taurine and hypotaurine metabolism, Steroid hormone biosynthesis, Arachidonic acid metabolism, and Alanine, aspartate, and glutamate metabolism (Figure 6H). From SMPDB analysis, the top pathways were the Retinol Metabolism, Estrone Metabolism, Pentose Phosphate Pathway, Malate-Aspartate Shuttle, and Pyrimidine Metabolism (Figure 6I). The top three enriched pathways included 28 metabolites, whose levels decreased in the M group compared to the control but were restored by AA intervention (Figure 6J). These findings suggest that AA alters and partially restores adenine-induced renal metabolic dysregulation, especially in the Retinol Metabolism, in CKD mice.

### 3.7 Effects of AA on the renal metabolism profiles in CKD mice may be related to gut microbiota

**Figure 7:**
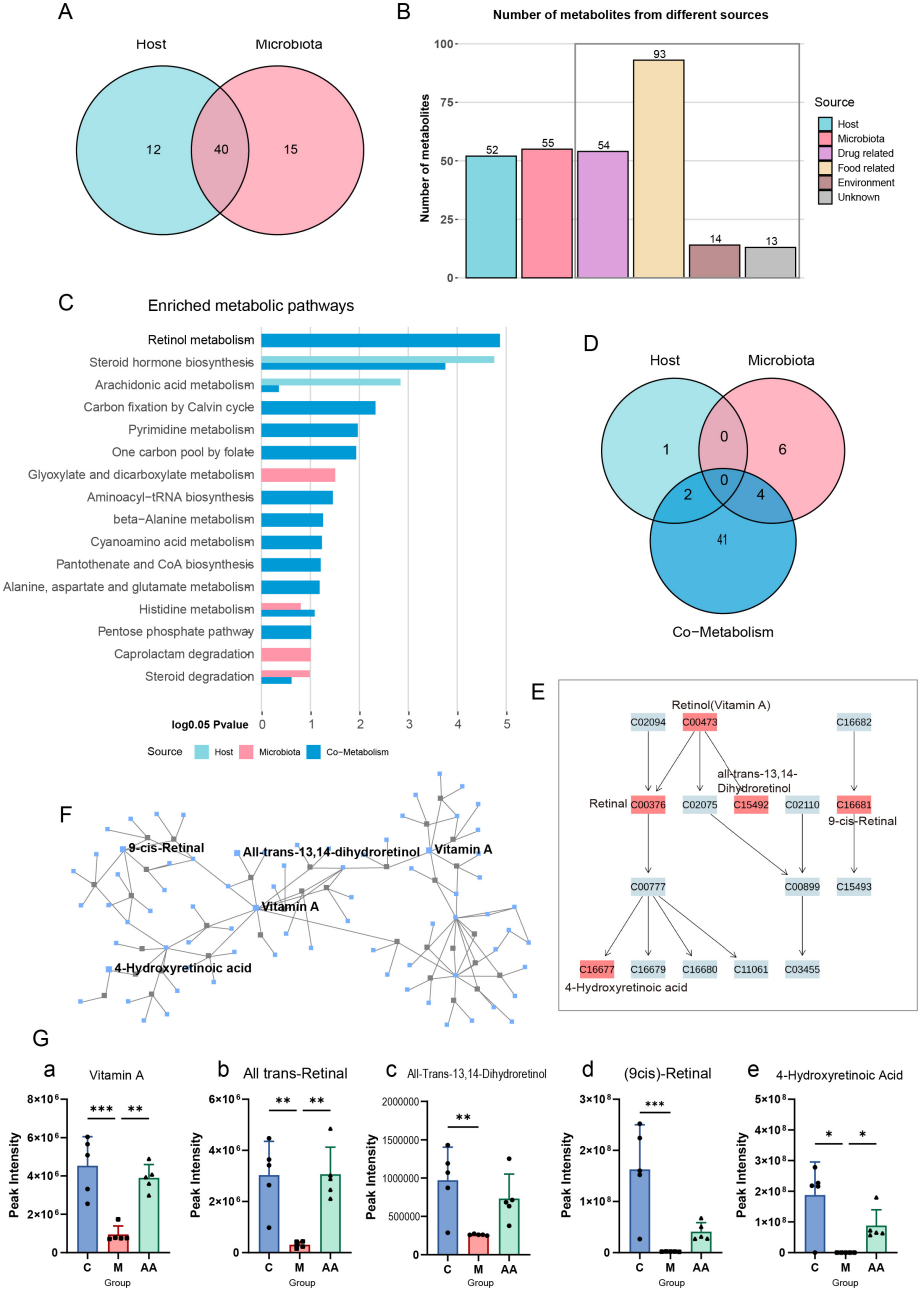
MetOrigin analysis results and in-depth analysis of Kidney Metabolism. **(A)** Venn diagram showing the origin of host-derived and microbiota-derived metabolites. **(B)** Column graph from MetOrigin analysis illustrating the originating sources of kidney metabolites. **(C)** Bar chart displaying *P*-values for metabolic pathways originating from the host, gut microbiota, or their interaction. **(D)** Venn diagram of KEGG pathway enrichment; the circles indicate the number of pathways specific to the source of metabolites. **(E)** KEGG pathway view for the enriched retinol metabolism pathway, with the KEGG ID shown in the box and significantly changing metabolites marked in red. **(F)** SMPDB pathway view of the enriched Retinol metabolism pathway, with enlarged blue squares indicating significantly changing metabolites. **(G)** Bar chart of peak intensity for five differential metabolites in Retinol metabolism. After normality testing, one-way ANOVA followed by Tukey's test or Kruskal–Wallis test followed by Dunn's test was used for multiple comparisons. AA, high dose AA at 30 mg/kg. *P < 0.05, **P < 0.01, *n* = 5.

In order to examine the origins of differential metabolites, we employed MetOrigin analysis to assess the potential influence of gut microbiota on the reprogramming of kidney metabolites. The overlapping differential metabolites from the two comparative groups were analyzed using the MetOrigin platform. The analysis of shared differential metabolites between two groups showed that 52 metabolites were host-derived, 55 originated from gut microbiota, and 40 were from both sources (Figure 7A). Among the 107 identified metabolites, those related to food constituted the largest proportion, totaling 93 metabolites (Figure 7B). Analysis showed that most enriched pathways resulted from microbiota-host co-metabolism, with 41 out of 48 pathways identified as such (Figure 7D). The top five enriched pathways were retinol metabolism, steroid hormone biosynthesis, arachidonic acid metabolism, carbon fixation by the Calvin cycle, and pyrimidine metabolism, with retinol metabolism being the most significant and impactful (Figure 7C). The KEGG (Figure 7E) and SMPDB (Figure 7F) analyses of differential metabolic pathways revealed that five metabolites enriched in the retinol metabolism pathway: vitamin A, retinal, all-trans-13,14-dihydroretinol, 9-cis-retinal and 4-hydroxyretinoic acid. These metabolites were significantly lower in the M group compared to the C group but increased significantly after AA intervention (Figures 7Ga–e), with 4-hydroxyretinoic acid (4-OH-RA) exhibiting the most significant up-regulation. It decreased 9.61-fold in the M group but increased 8.52-fold after AA treatment (Figure 7Ge and Supplementary Table S1). These findings suggest that the impact of AA on renal metabolites is likely associated with alterations in gut microbiota.

### 3.8 Correlation analysis of differential metabolites, gut microbiota, and effect indicators

**Figure 8:**
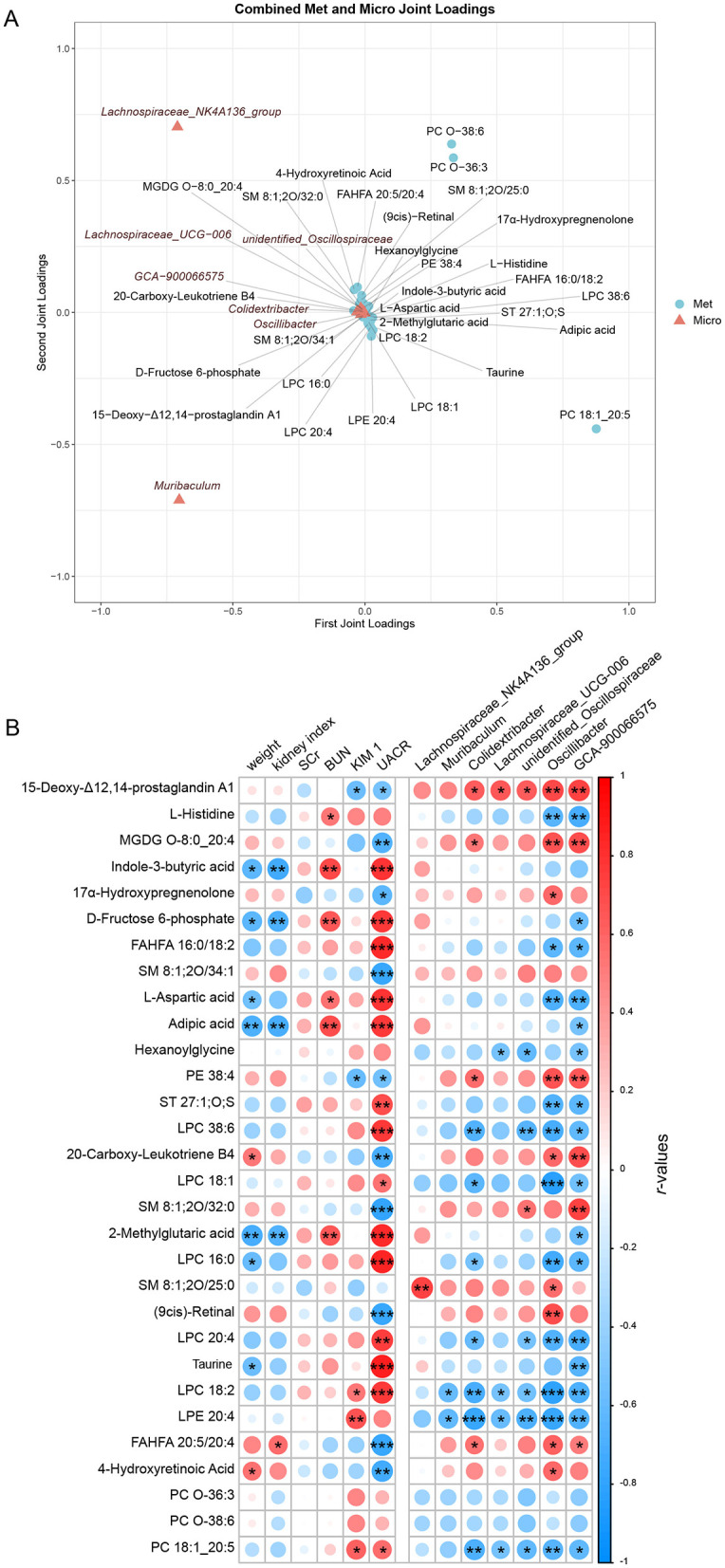
Correlation analyses of key differential metabolites, disease indicators, and bacterial genera. **(A)** O2PLS loading plot illustrating multi-omics associations between renal metabolomic profiles and microbial genera. Geometric symbols represent variables: circles denote metabolites, triangles indicate bacterial genera (italicized). **(B)** Heatmap of Spearman correlation analysis between critical metabolites and clinical indicators/bacterial taxa. AA, high dose AA at 30 mg/kg. Color gradient (red: positive; blue: negative) and symbol diameter proportionally represent correlation strength (*r*-values), with asterisks denoting statistical significance: *P < 0.05, **P < 0.01, ***P < 0.001.

To further explore the relationship between renal metabolic alterations, gut microbiota, and the protective effects on renal function following AA intervention, we conducted a correlational study that integrated renal metabolites, microbial profiles, and efficacy indicators. The integrated analysis was performed using the bidirectional O2PLS model, an unsupervised multivariate approach that quantifies global associations between two datasets while identifying key variables through loading value computation. Metabolites and microbial taxa with high loading values were considered critical contributors to inter-dataset similarity. As illustrated in Figure 8A, the gut microbiota exhibiting strongest correlations with renal metabolic changes were *Lachnospiraceae NK4A136 group* and *Muribaculum*, while phosphatidylcholine PC (18:1/20:5) emerged as the highest-weighted metabolite. Subsequent Spearman correlation analysis of the top 30 metabolites and 7 microbial genera revealed significant associations between 26 metabolites and disease indicators (Figure 8B). Notably, 24 metabolites showed significant correlations with UACR, with three demonstrating moderate-to-strong negative correlations: (9cis)-retinal (*r* = -−0.76, *P* < 0.001), 4-hydroxyretinoic acid (*r* = −0.72, *P* < 0.01), and 20-carboxy-leukotriene B4 (*r* = -−0.71, *P* < 0.01). Microbial correlation analysis demonstrated that all 26 metabolites were significantly associated with at least one key genus, particularly *Oscillibacter* and *GCA-900066575*, which showed associations with 20 and 22 metabolites, respectively (Supplementary Figure S2). These findings suggest that the identified metabolites may modulate critical metabolic pathways influencing host health, while highlighting the intricate interconnection between gut microbiota composition and host metabolism.

### 3.9 RNA-seq further illustrated the mechanisms of regulatory pathways associated with retinol metabolism

**Figure 9:**
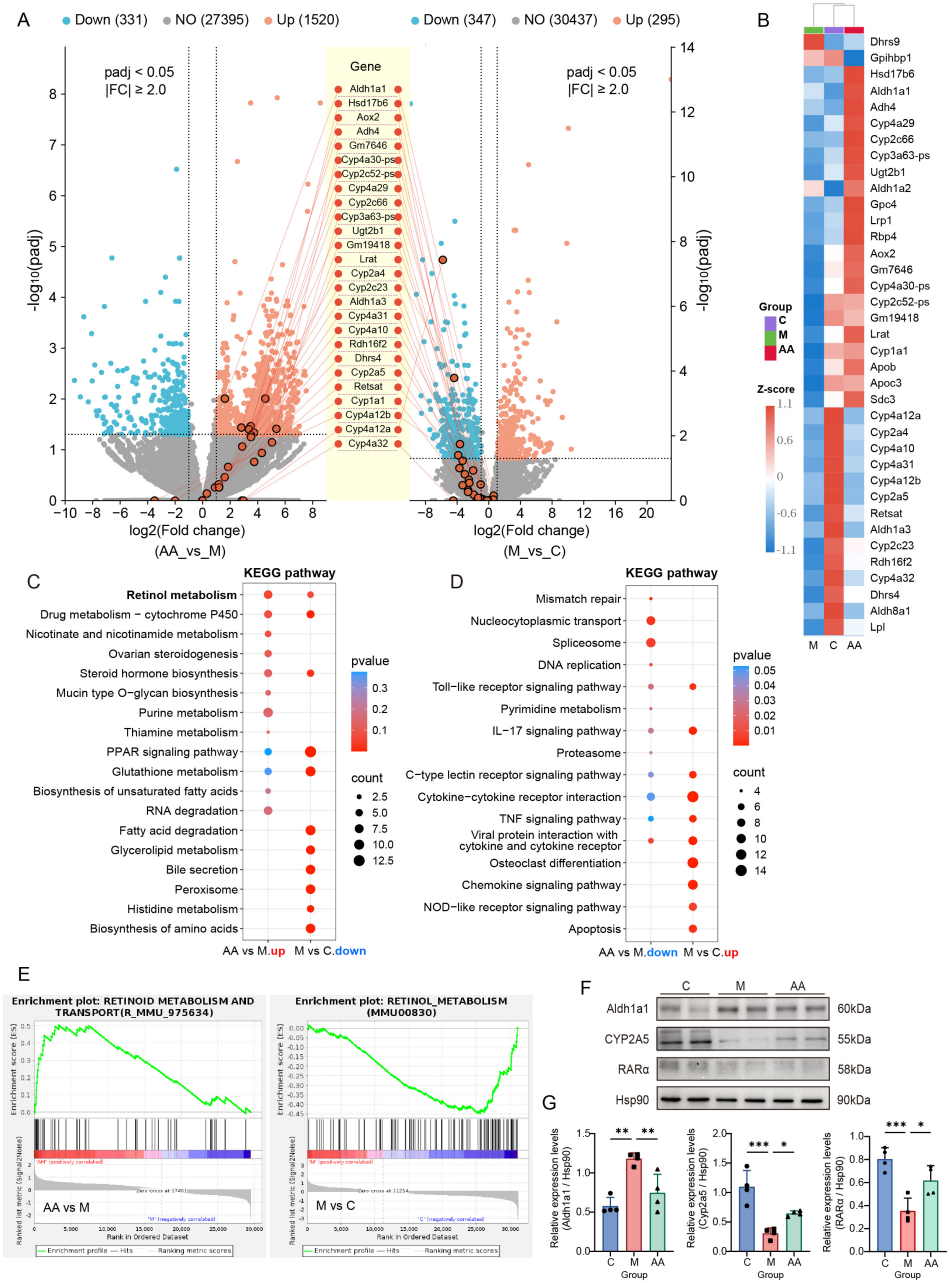
Transcriptomic analysis of kidney tissue revealing the effects of Asiatic acid on adenine-induced CKD model. **(A)** Volcano plots of DEGs. DEGs were filtered using stringent criteria of |log_2_ (FC)| ≥ 1 and *FDR* < 0.05. Key genes associated with retinol metabolism are highlighted. **(B)** Heatmap showing the expression levels (*Z*-score) of genes involved in the retinol metabolism pathway across the three groups. **(C)** KEGG enrichment analysis for pathways that were downregulated in M vs. C but upregulated following AA intervention. **(D)** KEGG enrichment analysis for pathways that were upregulated in M vs. C but downregulated by AA. **(E)** GSEA plots illustrating the enrichment of retinoid metabolism and transport pathways in AA vs. M and M vs. C comparisons. **(F, G)** Western blot analysis **(F)** and semi-quantification **(G)** of key enzymes (Aldh1a1, CYP2A5) and receptor (RARα) in the retinol metabolism pathway. Hsp90 served as the internal control. *n* = 5 for RNA-seq; *n* = 4 for Western blot. Following normality testing, one-way ANOVA with Tukey's test or Kruskal–Wallis with Dunn's test was used for multiple comparisons. AA, high dose AA at 30 mg/kg. *P < 0.05, **P < 0.01, ***P < 0.001.

Transcriptomic analysis of kidney tissue was conducted to investigate the impact of AA intervention on the adenine-induced CKD model compared to normal controls. The CKD model comparison identified 642 differentially expressed genes (DEGs; Figure 9A, right). Following AA intervention, 1,851 DEGs were identified, characterized by a significant upregulation of 1,520 genes in the AA group (Figure 9A, left). A cluster of genes critical for retinol metabolism was identified as being generally downregulated in the diseased kidney tissue but were upregulated in the therapeutic comparison (Figure 9B). Pathway analysis confirmed that AA robustly modulated several critical metabolic processes. Genes that were upregulated in the AA group were found to be highly enriched in retinol metabolism, drug metabolism—cytochrome P450, PPAR signaling pathway, and glutathione metabolism (Figure 9C). Conversely, pathways downregulated by the AA intervention were related to inflammation and proliferation processes, such as Toll-like receptor signaling pathway, TNF signaling pathway and IL-17 signaling pathway, that were initially upregulated in the CKD model (Figure 9D). Furthermore, Gene Set Enrichment Analysis (GSEA) confirmed retinol metabolism up and down regulation. In the AA-intervened group, the retinol metabolism-related pathway was up-regulated (Enrichment Score = 0.50, |*NES*| = 1.84), whereas it was down-regulated in the CKD group (Enrichment Score = −0.45, |*NES*| = 1.73, Figure 9E). The KEGG map (Supplementary Figure S3) visually confirmed that AA administration significantly modulated the expression of multiple key enzymes within the Retinol metabolism pathway, suggesting a potential mechanism for its protective effect in CKD. Aldehyde dehydrogenase 1A1 (ALDH1A1) catalyzes the conversion of retinal to retinoic acid and dehydrogenates 9-cis-retinal into 9-cis-retinoic acid (23–25). Human cytochrome p450 family 2 subfamily A member 6 (CYP2A6) catalyzes the oxidation of retinoic acid to 4-hydroxyretinoic acid, which orthologous is mouse Cyp2a5 (26). Its mRNA expression is induced by retinoic acid and downregulated during vitamin A depletion. Retinoic acid, 9-cis-retinoic acid, and hydroxyretinoic acid can bind to the nuclear receptor retinoid receptor (RAR/RXR). Transcriptomic analysis revealed that among RXR and RAR isoforms, only Rara exhibited significant mRNA upregulation. In contrast, Rxra, Rxrb, and Rxrg showed no significant transcriptional changes among groups, although a trend toward restoration of Rxra expression was noted in AA-treated mice (Supplementary Figure S4). Western blot analysis demonstrated that adenine-induced CKD mice exhibited significantly suppressed expression of ALDH1A1 and CYP2A5 in the mice kidneys, along with upregulated RARα expression. However, AA intervention alleviated these changes in key targets (Figures 9F, G).

### 3.10 Key metabolite involved in retinol metabolism attenuate fibrotic injury and oxidative stress in HK-2 cells

**Figure 10:**
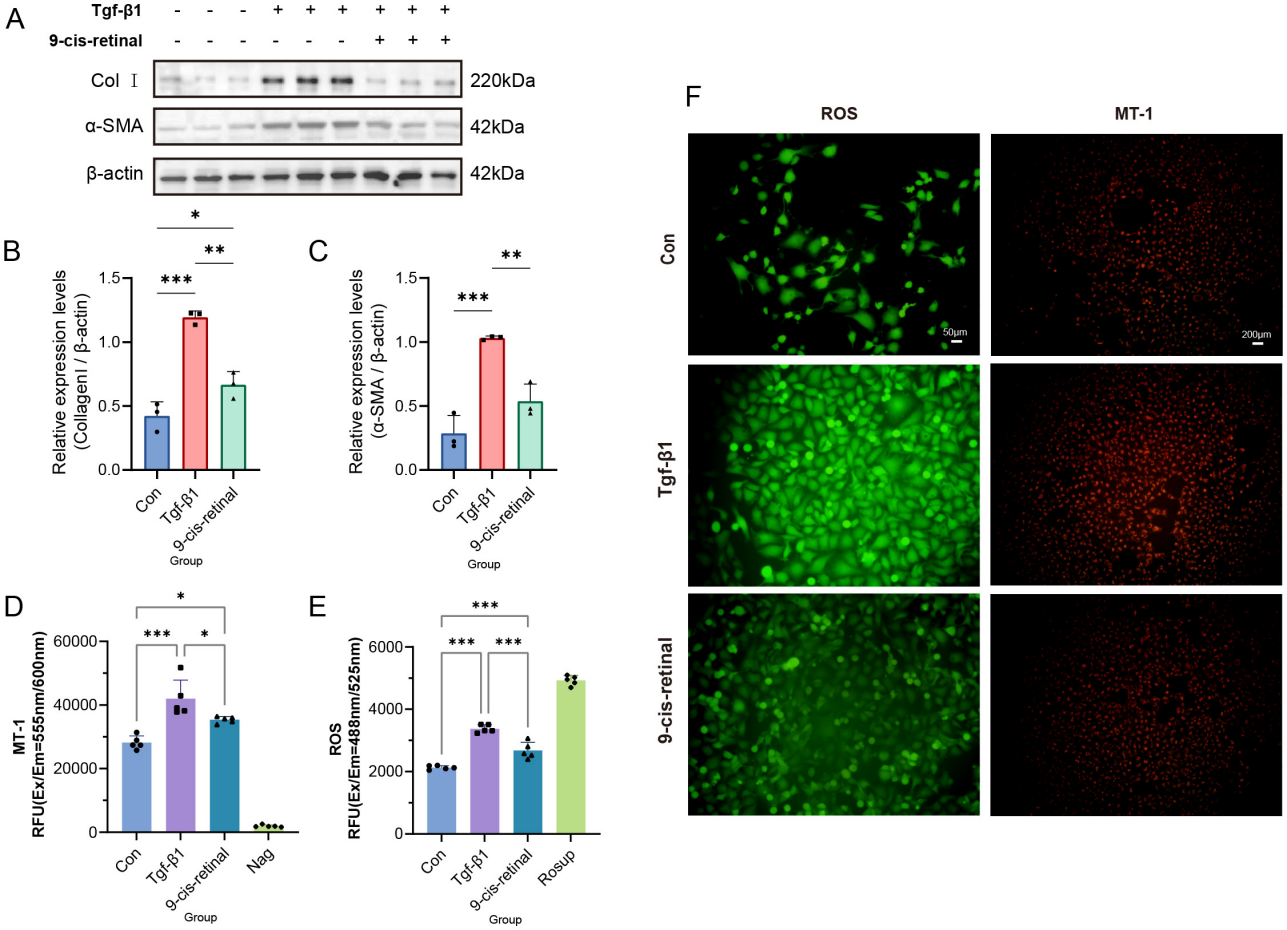
9-cis-retinal attenuate TGF-β1-induced fibrotic injury and oxidative stress in HK-2 cells. **(A–C)** Western blot analysis **(A)** and semi-quantification **(B, C)** of Collagen I (Col I) and α-SMA expression in HK-2 cells treated with TGF-β1 (10 ng/ml) and 9-cis-retinal (10 μM). **(D)** RFU bar chart for MT-1, Ex/Em = 555 nm/600 nm, *n* = 5. **(E)** RFU bar chart for ROS, Ex/Em = 488 nm/525 nm, *n* = 5. Rosup and Nag serve as positive/negative controls. **(F)** Representative fluorescent images of ROS (scale bar = 50 μm) and MT-1 (scale bar = 200 μm) staining. *P < 0.05, **P < 0.01, ***P < 0.001.

Retinoids play a crucial role in the repair of renal tubular injury (23), which led us to select human proximal tubule epithelial cells (HK-2) for our *in vitro* investigation (27). Among the retinol metabolism metabolites restored by AA, 9-cis-retinal showed the strongest negative correlation with UACR. Our findings revealed that 9-cis-retinal significantly suppressed TGF-β1-induced upregulation of fibrotic markers, including Col I and α-SMA (Figures 10A–C). Furthermore, 9-cis-retinal treatment effectively reduced cytosolic ROS production and restored MMP in injured HK-2 cells (Figures 10D–F), thereby demonstrating the protective effect of retinoids on renal tissue following fibrotic injury and oxidative stress. In addition, 4-OH-RA—the most strongly upregulated retinoid following AA intervention—also showed protective effects in HK-2 cells; these data are presented in Supplementary Figure S5. Next, we investigated whether AA itself exerts direct renoprotective effects. Direct administration of AA significantly attenuated TGF-β1-induced oxidative stress and fibrotic marker expression. The protective effects of AA on Col I/α-SMA expression and ROS/MMP levels were partially abolished by the addition of Methoxsalen, a CYP2A5/6 inhibitor (Supplementary Figure S6). It is noteworthy that although AA demonstrated direct protective effects on tubular cells *in vitro*, consistent with previous reports highlighting AA's anti-fibrotic and antioxidant potential (22, 28), its oral administration results in low bioavailability and limited kidney accumulation.

## 4 Discussion

Metabolic dysregulation functions bidirectionally as both a driver and consequence of renal fibrosis (5), a pathophysiological process co-modulated by host-microbiota metabolic crosstalk. Emerging evidence has highlighted the regulatory role of gut microbiota in renal pathophysiology (29). Integrated multi-omics approaches combining genomic sequencing with metabolomic profiling offer novel translational strategies for microbiome-targeted nephropathy interventions (30). AA, a natural product with multi-target renoprotective effects, shows promise in treating acute and chronic kidney injuries by protecting tubular epithelial cells, stabilizing podocytes, and exhibiting antioxidant and anti-fibrotic activities. This study revealed that AA intervention significantly attenuated the decline of renal function in adenine-induced CKD mice, as evidenced by reduced Scr, BUN, and UACR, concomitant with suppressed Col-I, α-SMA, and TGF-β1 expression, along with diminished renal collagen deposition. Renal metabolomic analysis revealed that AA principally modulates key metabolic pathways, including retinol metabolism, steroid hormone biosynthesis, and arachidonic acid metabolism. MetOrigin-based metabolic tracing further demonstrated that this metabolic reprogramming depended on host-microbiota co-metabolism. Notably, AA administration prevented adenine-induced gut dysbiosis, enriching beneficial taxa including *Lachnospiraceae NK4A136 group, Muribaculum*, and *Clostridium sensu stricto 1* while suppressing pathogenic bacterial proliferation. Collectively, our findings establish that AA alleviates CKD progression may through dual mechanisms restoring gut microbiota homeostasis and remodeling renal metabolic networks. The core therapeutic mechanism involves targeted modulation of the gut-kidney axis, suggesting its potential as a versatile treatment for renal protection and reducing fibrosis.

Renal metabolomic analysis demonstrated that AA intervention altered retinol metabolism, with MetOrigin-based metabolic tracing identifying this pathway as the most critical node in host-microbiota interactions. Retinol (vitamin A) and its bioactive derivatives (retinoids) play essential roles in maintaining renal homeostasis and facilitating tubular repair in adults, while being indispensable for embryonic kidney development (23). Notably, enhanced retinol intake has been associated with reduced CKD risk in type 2 diabetic populations (31). Our findings further revealed diminished renal retinol/retinoid levels in fibrotic kidneys, consistent with prior clinical observations. Under physiological conditions, hepatic-stored retinol is transported to the kidneys via retinol-binding protein 4, which undergoes glomerular filtration followed by proximal tubular reabsorption and catabolism to release retinol. However, impaired renal filtration and reabsorption capacities in CKD likely drive intrarenal retinol depletion (32). The elevated oxidative stress in CKD may also promote non-enzymatic oxidation of retinoids, further contributing to intrarenal retinol depletion (33). Metabolically, retinol undergoes oxidation to bioactive retinaldehyde-a reversible conversion-before being irreversibly catalyzed by ALDH1A isozymes (ALDH1A1/ALDH1A2/ALDH1A3) into retinoic acid (RA), representing the rate-limiting step in retinoid metabolism (34). As an endogenous agonist for retinoic acid receptors (RARα/β/γ), RA exerts anti-fibrotic and pro-regenerative effects. Experimental evidence indicates that RARα deficiency triggers mitochondrial dysfunction, apoptosis, and failed tubular repair (35). Although no significant transcriptional changes in RXR isoforms were observed, their activity may still be modulated post-transcriptionally or by endogenous ligands. Notably, 9-cis-retinal, which functions as an antagonist of RXR/PPARγ (36), was restored by AA and negatively correlated with UACR. The reduced expression of Col-I and α-SMA in AA-treated mice is consistent with enhanced retinoid signaling, as these genes are negatively regulated by this pathway (37). Together with the increase in 4-hydroxyretinoic acid (a potential RAR agonist), these observations suggest that AA may exert its renoprotective effects through multiple mechanisms along the retinoid axis (34).

Beyond retinol reprogramming, integrative pathway analysis identified steroid hormone biosynthesis and arachidonic acid metabolism as additional regulatory nodes of AA intervention. In the steroidogenic cascade, adenine-induced injury precipitated a critical depletion of 17α-hydroxypregnenolone, a pivotal precursor governing downstream glucocorticoid and sex hormone synthesis (38). AA treatment effectively normalized levels of cortisol, testosterone, and estradiol, which had been systemically suppressed in the CKD model. The restoration of these mediators likely contributes to the stabilization of glomerular hemodynamics and suppression of fibrotic signaling (39, 40). Notably, the observed suppression of sex hormone levels in male adenine-model mice phenocopies findings from female-specific renal models (41), validating this model's translational relevance across sex-specific pathophysiology. Within the arachidonic acid metabolic axis, AA promoted a shift toward an anti-inflammatory microenvironment by modulating the COX/LOX pathways. Specifically, AA upregulated 15-deoxy-Δ^12, 14^-prostaglandin J2 (15d-PGJ2) and 20-carboxy-LTB4. These mediators serve to activate PPARγ (16) and antagonize neutrophil-mediated inflammatory injury, respectively (42–44). These coordinated actions demonstrate AA's capacity to rebalance proinflammatory and anti-inflammatory mediators, synergistically ameliorating lipid metabolic dysregulation and improving renal microenvironmental homeostasis.

Dysbiosis of intestinal flora is one of the key drivers of the pathological mechanisms of CKD (45). LDA revealed *Ruminococcaceae, Lachnospiraceae*, and *Clostridia* as predominant bacterial families enriched following AA intervention. It was found that the abundance of *Ruminococcaceae bromii* decreased significantly with the progression of CKD, suggesting that this bacterium may delay disease progression by attenuating renal fibrosis (8). Notably, AA-induced abundance changes in Lachnospiraceae and Clostridia was consistent with the results of a previous study examining the common features of gut flora in animals with experimental kidney disease (46), further supporting their generalized role in the regulation of CKD pathology. At the genus level, Kruskal–Wallis test analysis revealed that *Muribaculum, Lachnospiraceae NK4A136 group*, and *Bifidobacterium* were significantly altered among groups. Notably, *Lachnospiraceae NK4A136_group, Muribaculum*, and *Clostridium sensu stricto 1* exhibited significant enrichment in the AA-treated group. O2PLS analysis also confirmed these taxa as core discriminative genera. Functionally, these bacteria share metabolic competence in fermenting complex polysaccharides to produce short-chain fatty acids (SCFAs), including butyrate and acetate, which collectively enhance intestinal barrier integrity, suppress pathogenic overgrowth, and regulate host energy homeostasis (47–49).

At the species level, AA intervention significantly enriched *Lactobacillus intestinalis (L. intestinalis), Clostridium sp*., and *Mucispirillum schaedleri (M. schaedleri)*. The probiotic *L. intestinalis* exhibits ALDH activity, synergizing with host ALDH1A2 to enhance RA biosynthesis. This microbial-host co-metabolism activates intestinal RARα-mediated anti-inflammatory responses (50). *Clostridium sp*. demonstrates dual metabolic functions: purine degradation capacity (51) and bile acid biotransformation through conversion of chenodeoxycholic acid to ursodeoxycholic acid (52). The resultant ursodeoxycholic acid activates bile acid signaling pathways to ameliorate mitochondrial dysfunction and protect renal tubules (53). Although *M. schaedleri* predominantly colonizes the murine intestinal mucus layer as a core commensal in laboratory mice rather than humans (54), its genomic repertoire encodes specialized oxygen/ROS scavenging systems (55), suggesting potential regulatory roles in inflammatory modulation. *Parabacteroides gordonii* is positively correlated with vascular risk factors (56), yet its abundance declines in hypertensive renal injury models (57). While the functional implications remain to be fully elucidated, the elevated abundance of *Parabacteroides gordonii* in CKD mice was mitigated by AA treatment, hinting at a modest role in the gut microbial shifts accompanying renal dysfunction. The above results suggest that AA provides a new microbial target for CKD treatment by remodeling the structure of intestinal flora, enhancing the function and metabolic activity of beneficial bacteria, and synergistically ameliorating renal inflammation and metabolic disorder.

Previous studies indicate that AA content in fresh canella asiatica ranges from is 0.18–0.19 mg/g, making these doses achievable through dietary supplements of *Centella asiatica* extract (58). Due to its poor aqueous solubility and rapid metabolism, AA exhibits low oral bioavailability (59). Strategies to enhance its durability and bioavailability remain largely unexplored. As a highly lipophilic molecule, AA is likely eliminated via hepatobiliary excretion and subsequently excreted predominantly through the fecal route (60). Its renoprotective effects may be mediated, at least in part, by gut microbiota-derived metabolites, as supported by our antibiotic depletion experiments. Future pharmacokinetic studies in patients with CKD are warranted to establish optimal dosing regimens.

While this study provides novel insights into the mechanisms of AA in alleviating adenine-induced CKD through the gut-kidney axis, several limitations should be acknowledged. First, the experimental design utilized a single murine model and focused exclusively on male mice. Although the consistency of steroid hormone alterations across genders supports the robustness of the model, future studies incorporating female cohorts and alternative CKD models are necessary. Second, our antibiotic depletion experiment demonstrates a strong correlation between the gut microbiota and the renoprotective effects off AA, supporting the necessity of the gut microbiota in mediating these benefits. To further confirm the gut-kidney axis, the fecal microbiota transplantation (FMT) using germ-free animal models can be employed in future studies. Although studies indicate that using 1% DMSO as a solvent for insoluble compounds is generally safe, it may cause mild gastric irritation or mucosal damage. To address this, chemical structure modifications could be undertaken to enhance the solubility of AA in future. Third, the sample size is relatively limited for high-dimensional multi-omics analyses, which may reduce statistical power and increase the risk of false-positive results. Future studies with larger cohorts are warranted to validate the generalizability of these findings. Fourth, the dose-response effects of AA were partially explored, but detailed pharmacokinetic studies and long-term safety assessments are warranted before clinical translation. Finally, the functional roles of specific microbial taxa (e.g., *Lachnospiraceae NK4A136 group* and *Mucispirillum schaedleri*) in retinol or arachidonic acid metabolism require further mechanistic validation using *in vitro* co-culture systems or genetically engineered strains.

## 5 Conclusion

In summary, this study demonstrates that AA ameliorates renal injury and fibrosis in adenine-induced CKD mice. Antibiotic depletion experiments indicate that an intact gut microbiota is required for the renoprotective effects of AA, supporting a functional association along the gut–kidney axis. Integrated multi-omics analysis further reveals that AA administration is associated with remodeling of the gut microbiota and reprogramming of renal retinol metabolism. These findings suggest that AA may serve as a dietary-derived modulator of the gut–kidney axis with potential benefits for kidney health.

## Data Availability

The original contributions presented in the study are included in the article/Supplementary material. The raw data of metabolomics and RNA-seq have been deposited in the MetaboLights (MTBLS14232) and NCBI GEO (GSE328634) databases.
