# Supplementary material for: Asiatic acid from Centella asiatica alleviates renal fibrosis: coordinated modulation of the gut–kidney axis and retinol metabolism
Source: Front Nutr. 2026 Jul 10;13:1802674. doi: 10.3389/fnut.2026.1802674 (PMC13395619; doi:10.3389/fnut.2026.1802674)
Supplement: Supplementary file 1 [file Presentation_1.pptx]

## Slide 1
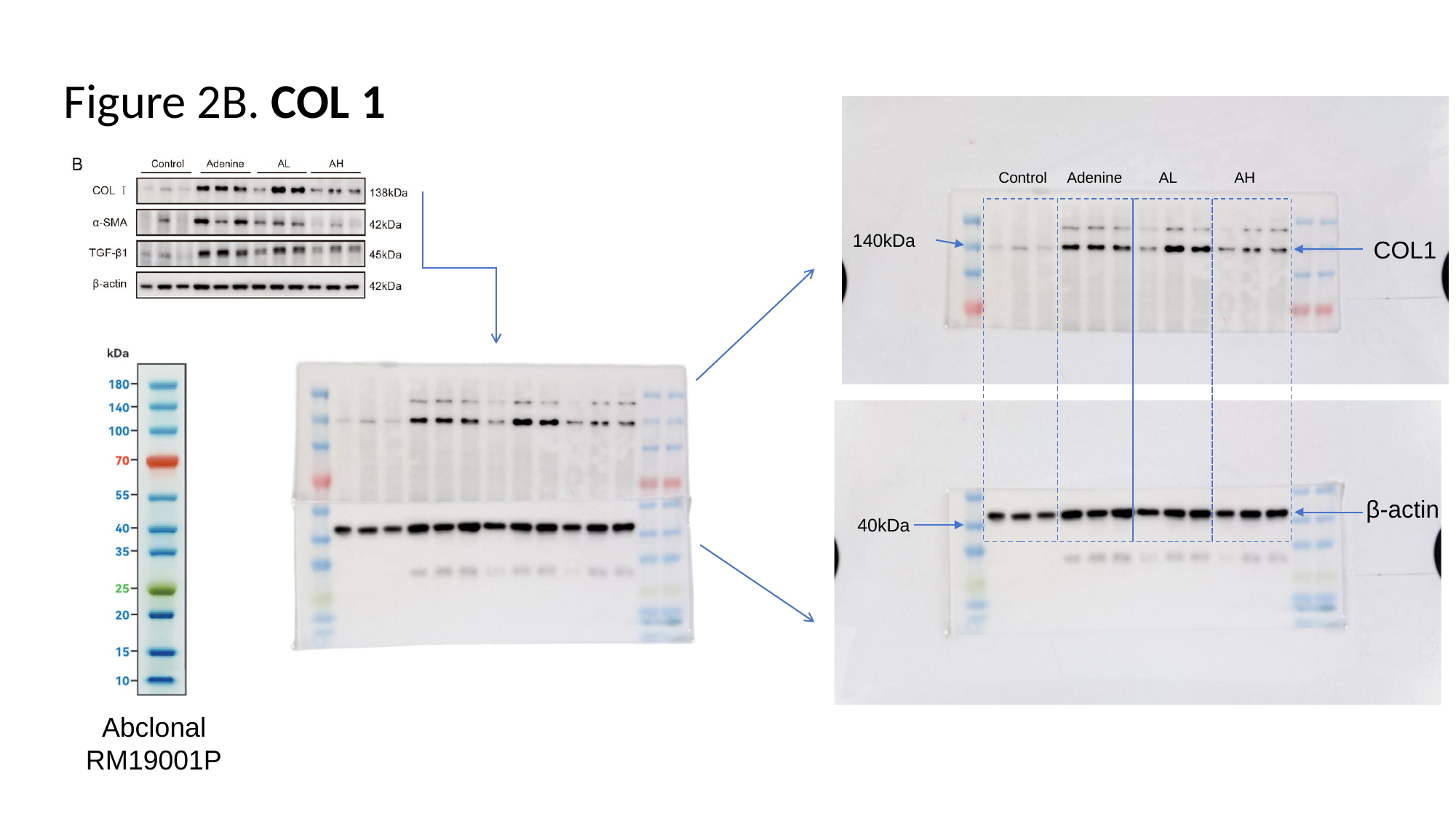

# Figure 2B. COL 1
Control Adenine AL AH
140kDa
COL1
β-actin
40kDa
Abclonal
RM19001P

## Slide 2
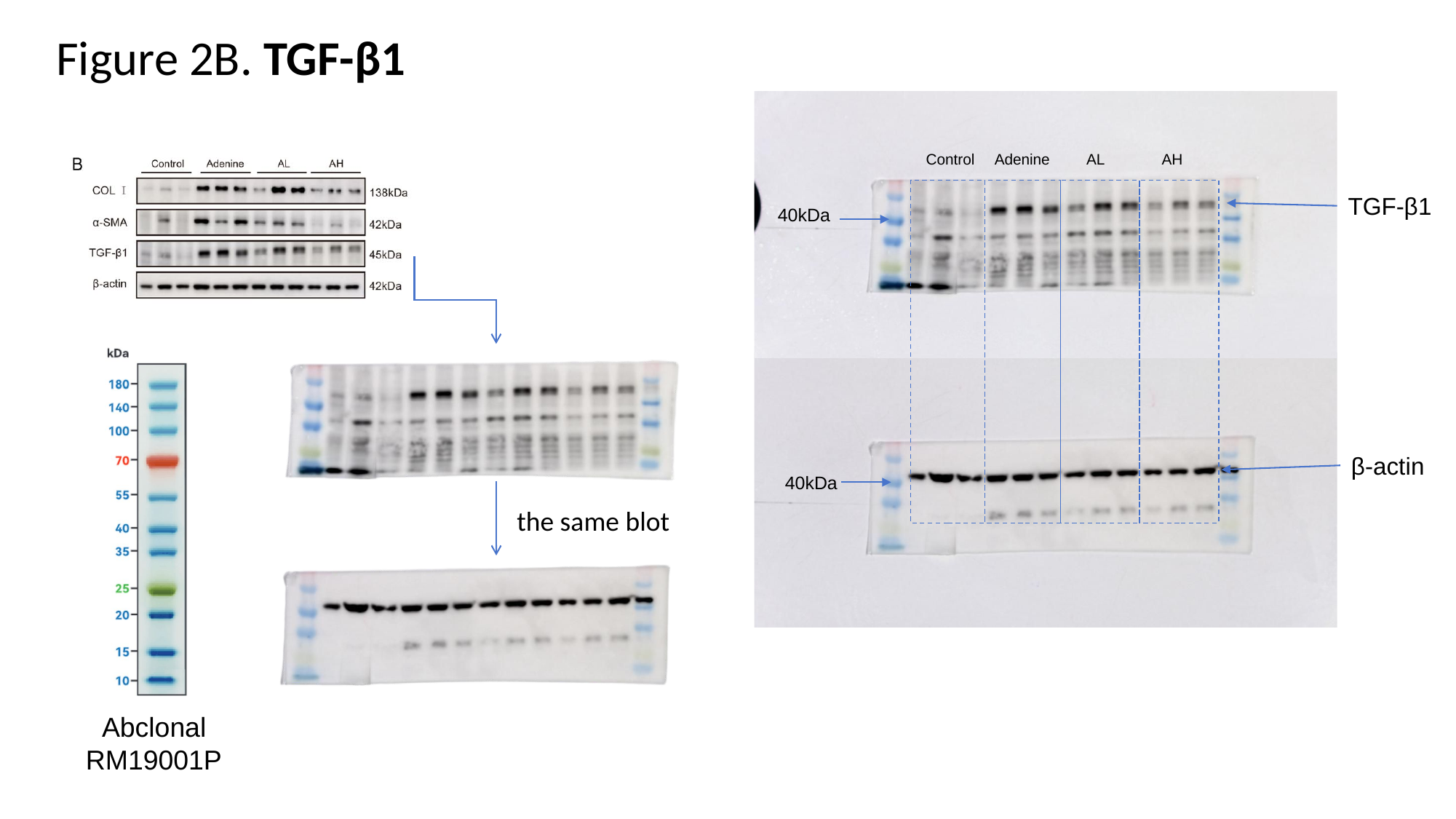

Figure 2B. TGF-β1
Control Adenine AL AH
TGF-β1
40kDa
β-actin
40kDa
the same blot
Abclonal
RM19001P

## Slide 3
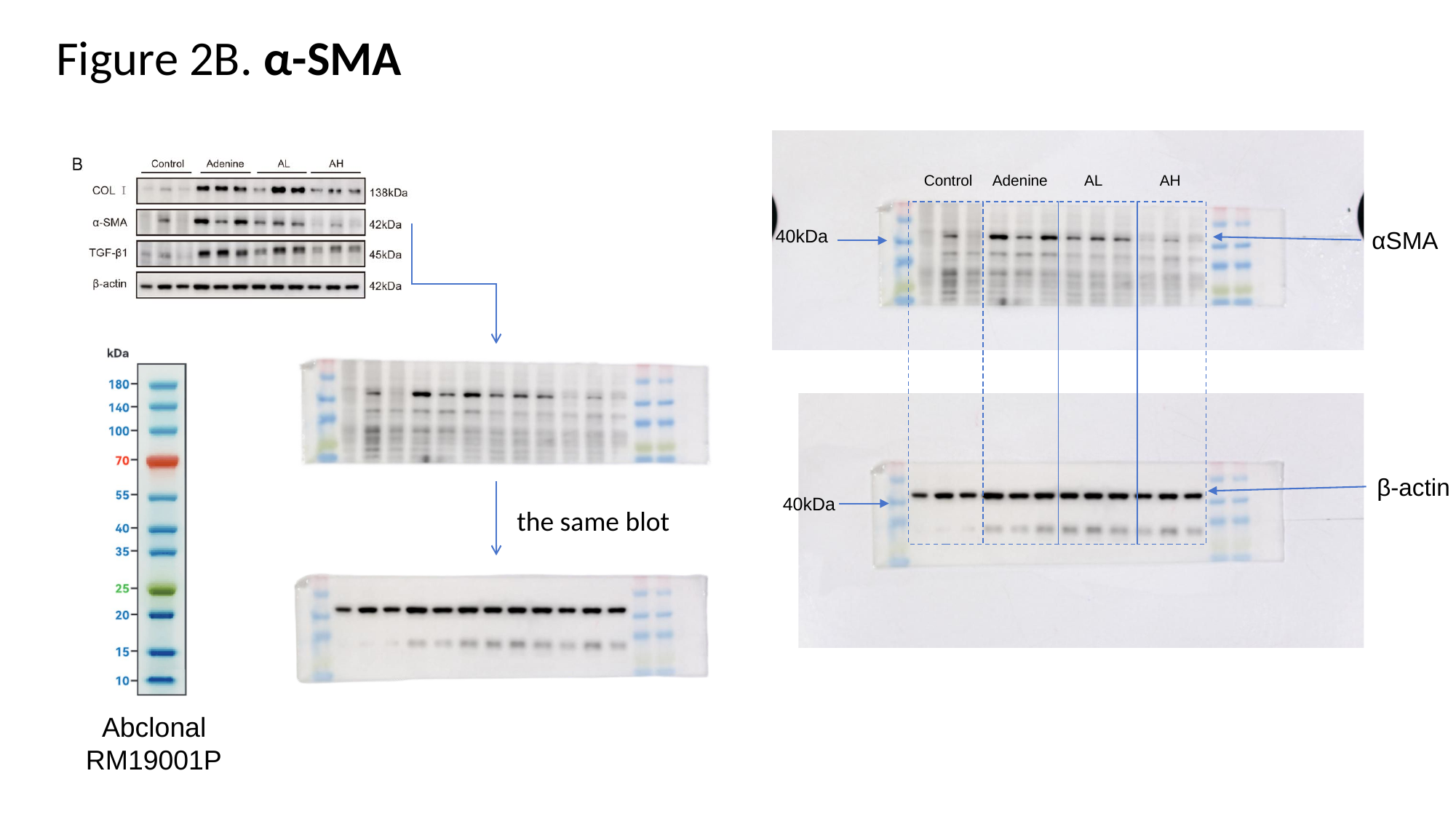

Figure 2B. α-SMA
Control Adenine AL AH
40kDa
αSMA
β-actin
40kDa
the same blot
Abclonal
RM19001P

## Slide 4
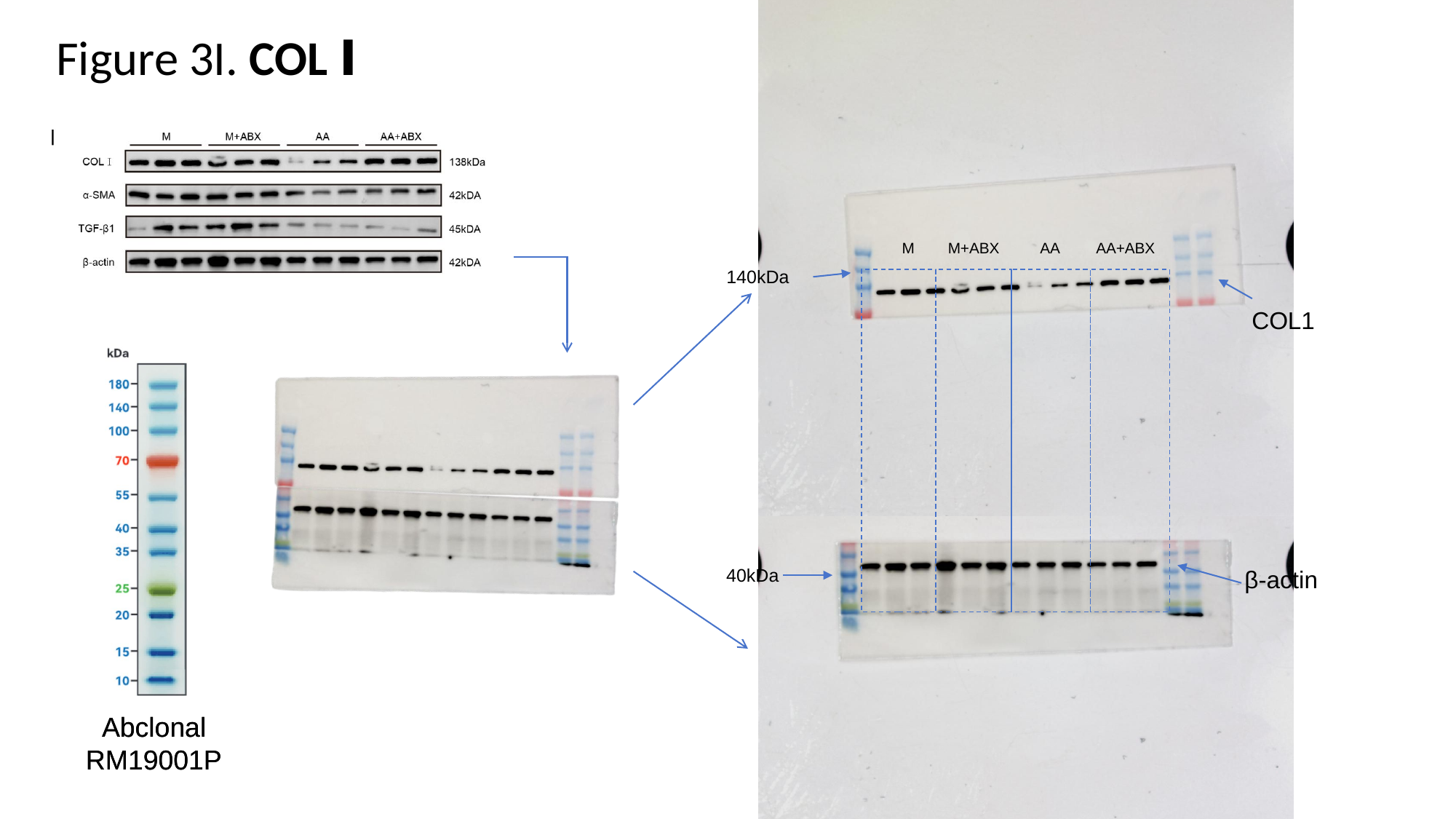

Figure 3I. COL Ⅰ
 M M+ABX AA AA+ABX
140kDa
COL1
40kDa
β-actin
Abclonal
RM19001P
Abclonal
RM19001P

## Slide 5
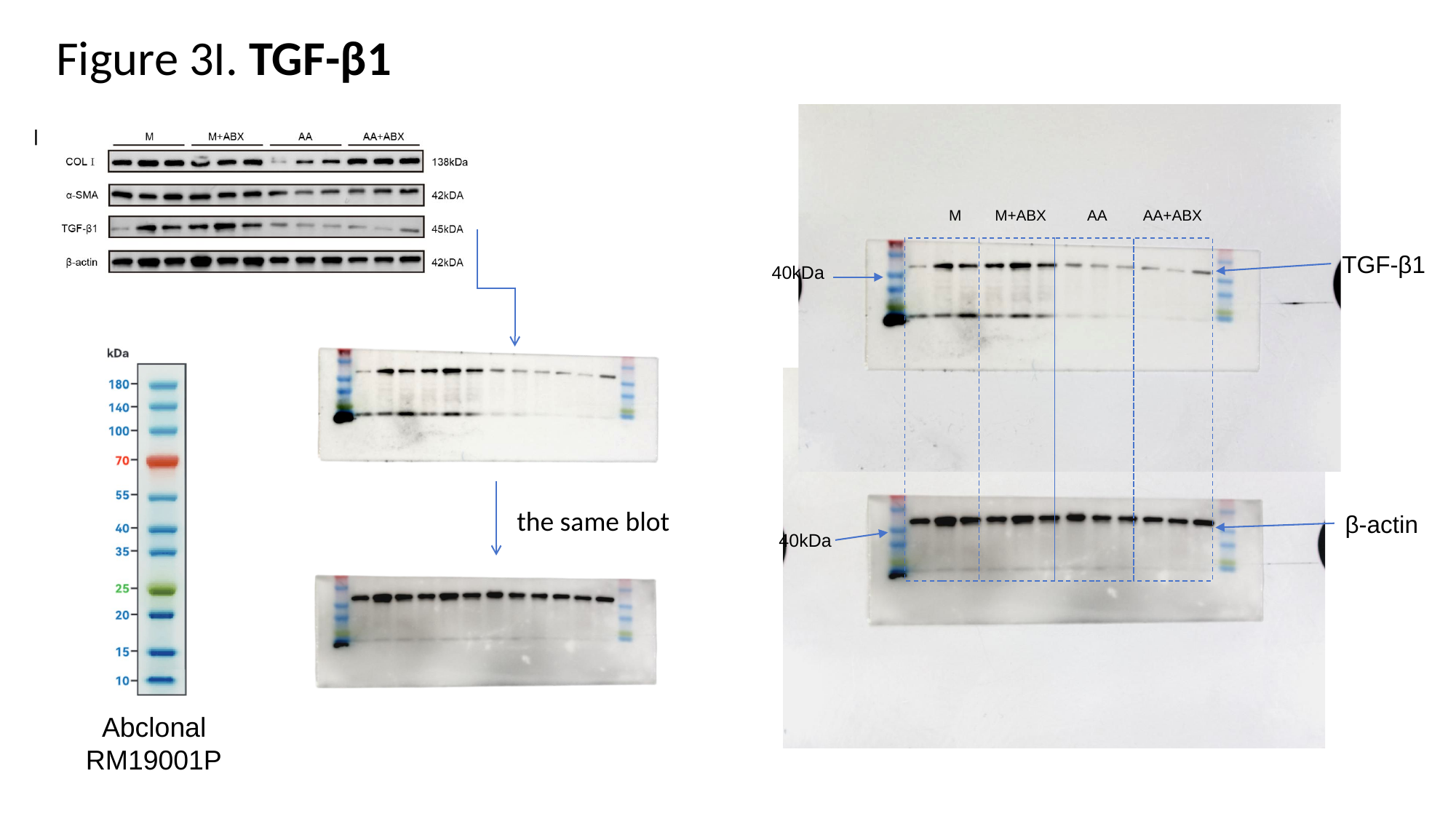

Figure 3I. TGF-β1
 M M+ABX AA AA+ABX
TGF-β1
40kDa
the same blot
β-actin
40kDa
Abclonal
RM19001P

## Slide 6
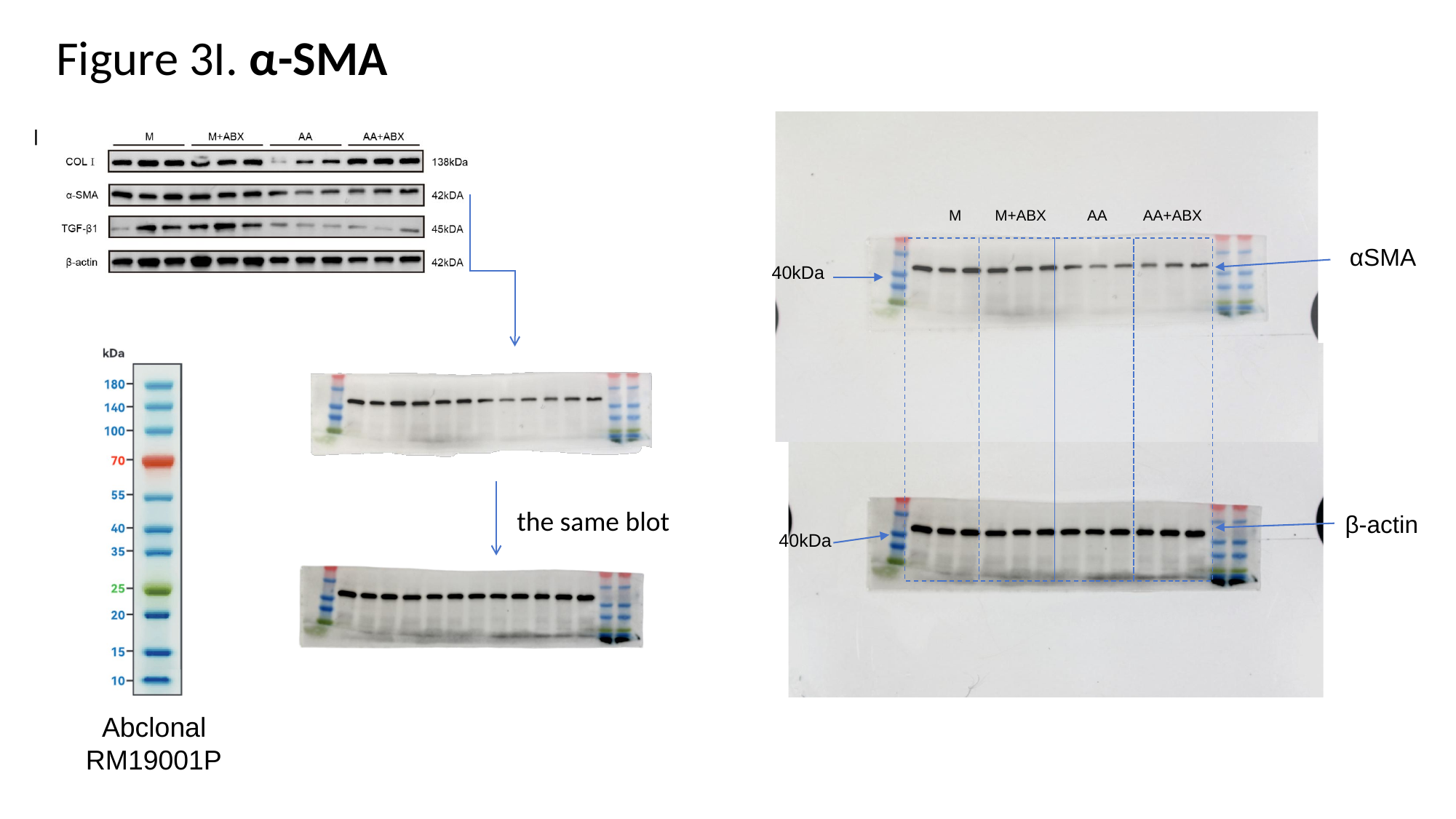

Figure 3I. α-SMA
 M M+ABX AA AA+ABX
αSMA
40kDa
the same blot
β-actin
40kDa
Abclonal
RM19001P

## Slide 7
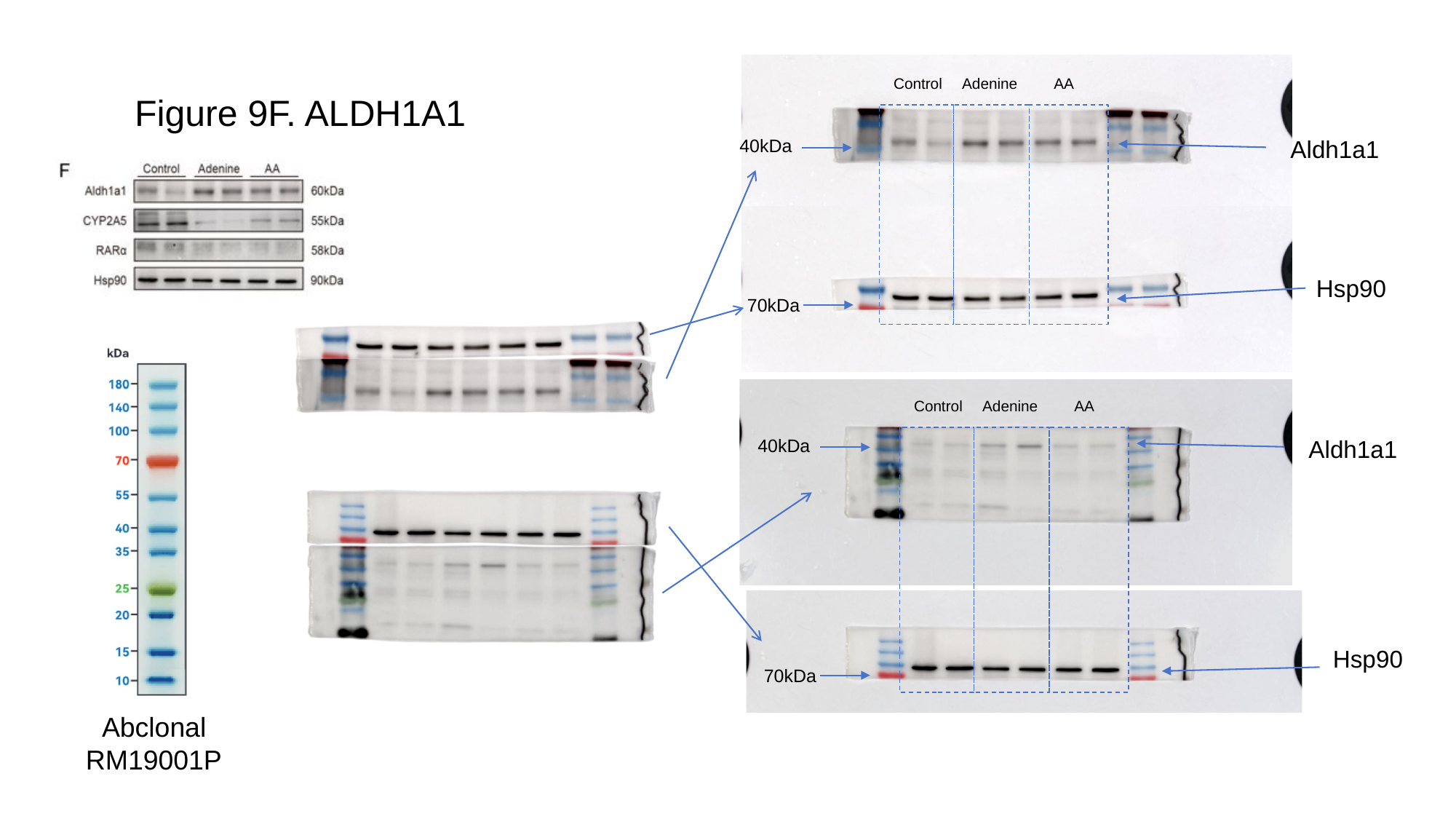

# Figure 9F. ALDH1A1
Control Adenine AA
40kDa
Aldh1a1
Hsp90
70kDa
Control Adenine AA
40kDa
Aldh1a1
Hsp90
70kDa
Abclonal
RM19001P

## Slide 8
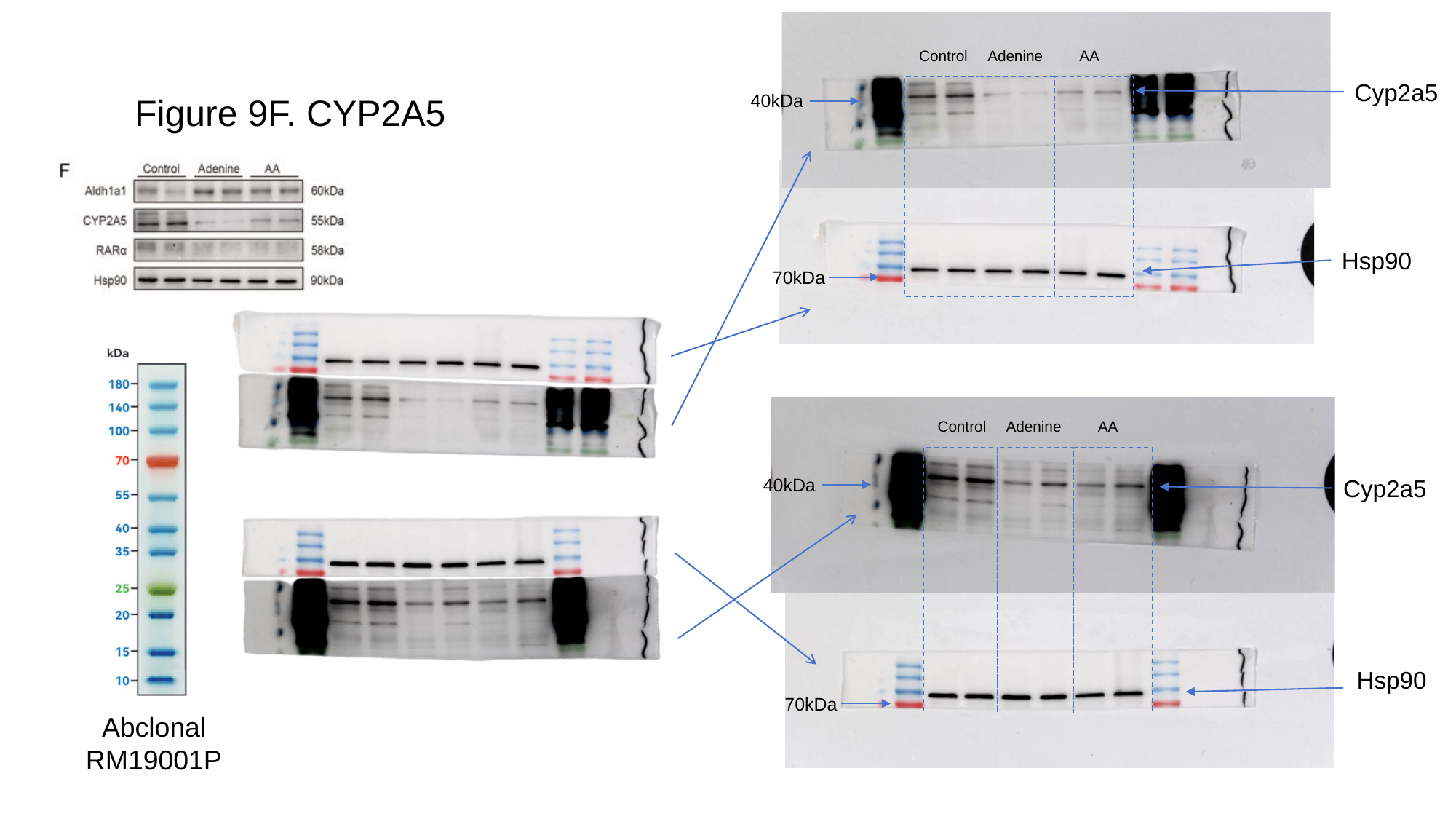

# Figure 9F. CYP2A5
Control Adenine AA
Cyp2a5
40kDa
Hsp90
70kDa
Control Adenine AA
40kDa
Cyp2a5
Hsp90
70kDa
Abclonal
RM19001P

## Slide 9
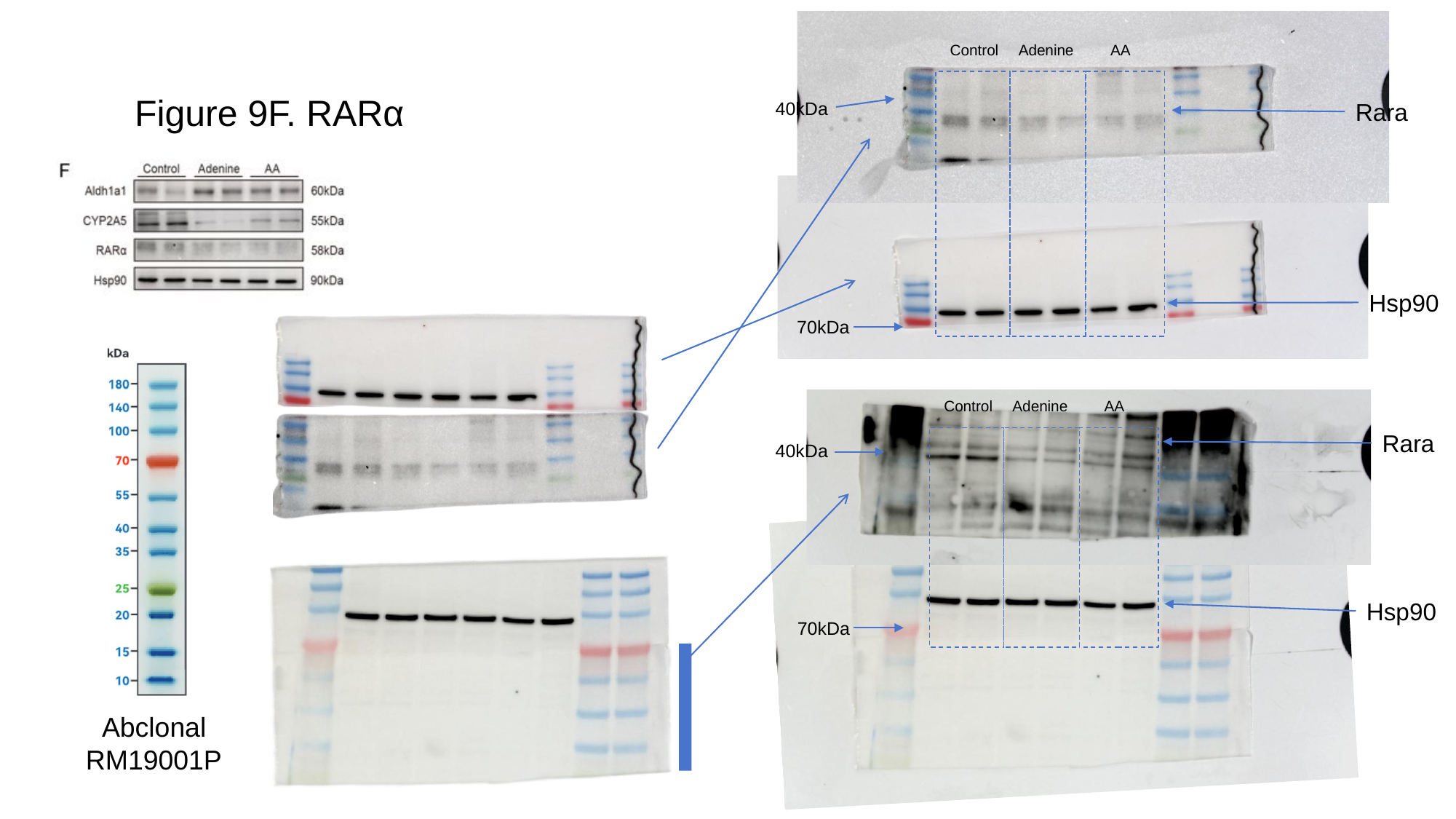

# Figure 9F. RARα
Control Adenine AA
40kDa
Rara
Hsp90
70kDa
Control Adenine AA
Rara
40kDa
Hsp90
70kDa
Abclonal
RM19001P

## Slide 10
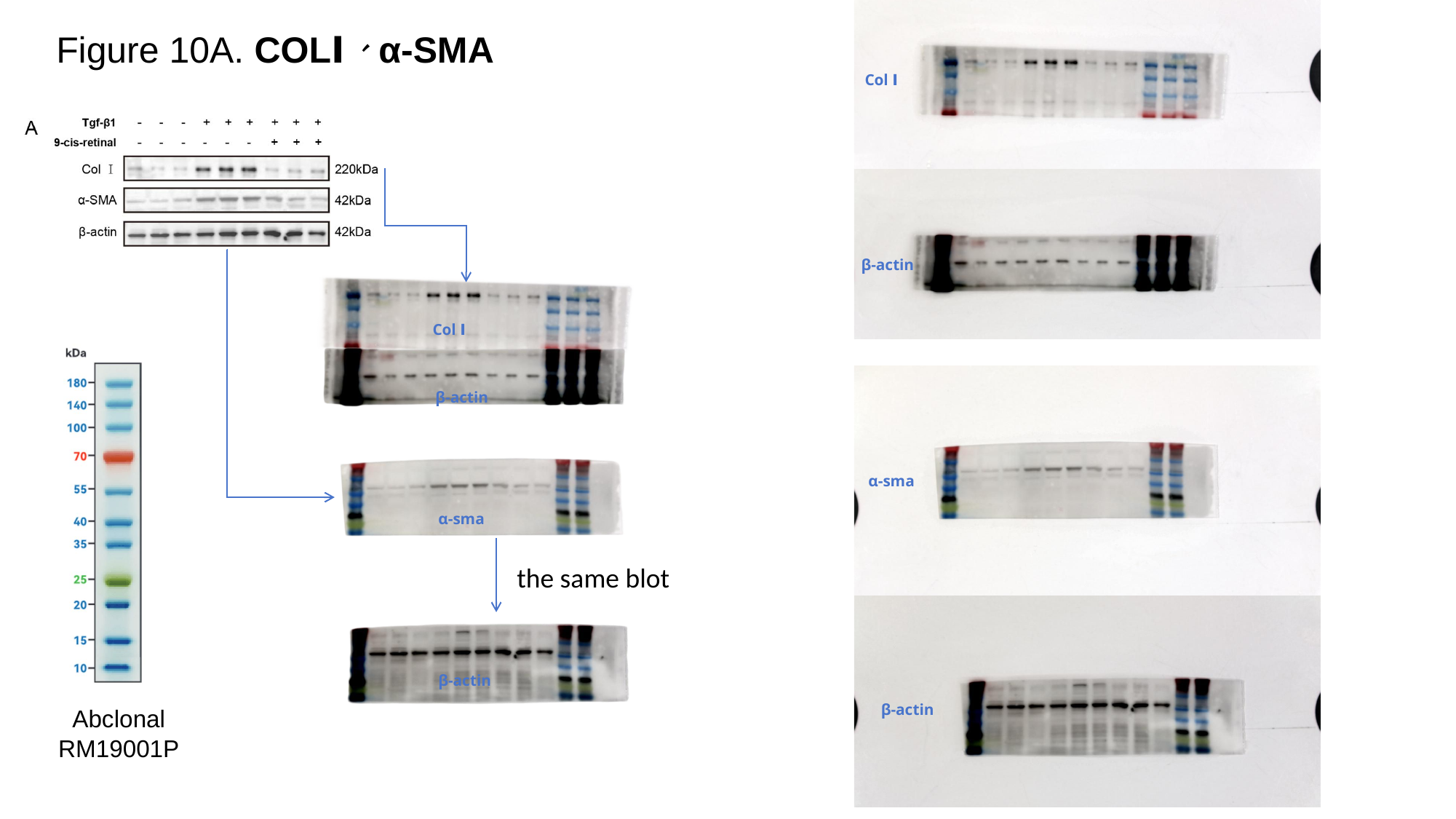

Figure 10A. COLⅠ、α-SMA
Col Ⅰ
β-actin
Col Ⅰ
β-actin
α-sma
α-sma
the same blot
β-actin
β-actin
Abclonal
RM19001P

## Slide 11
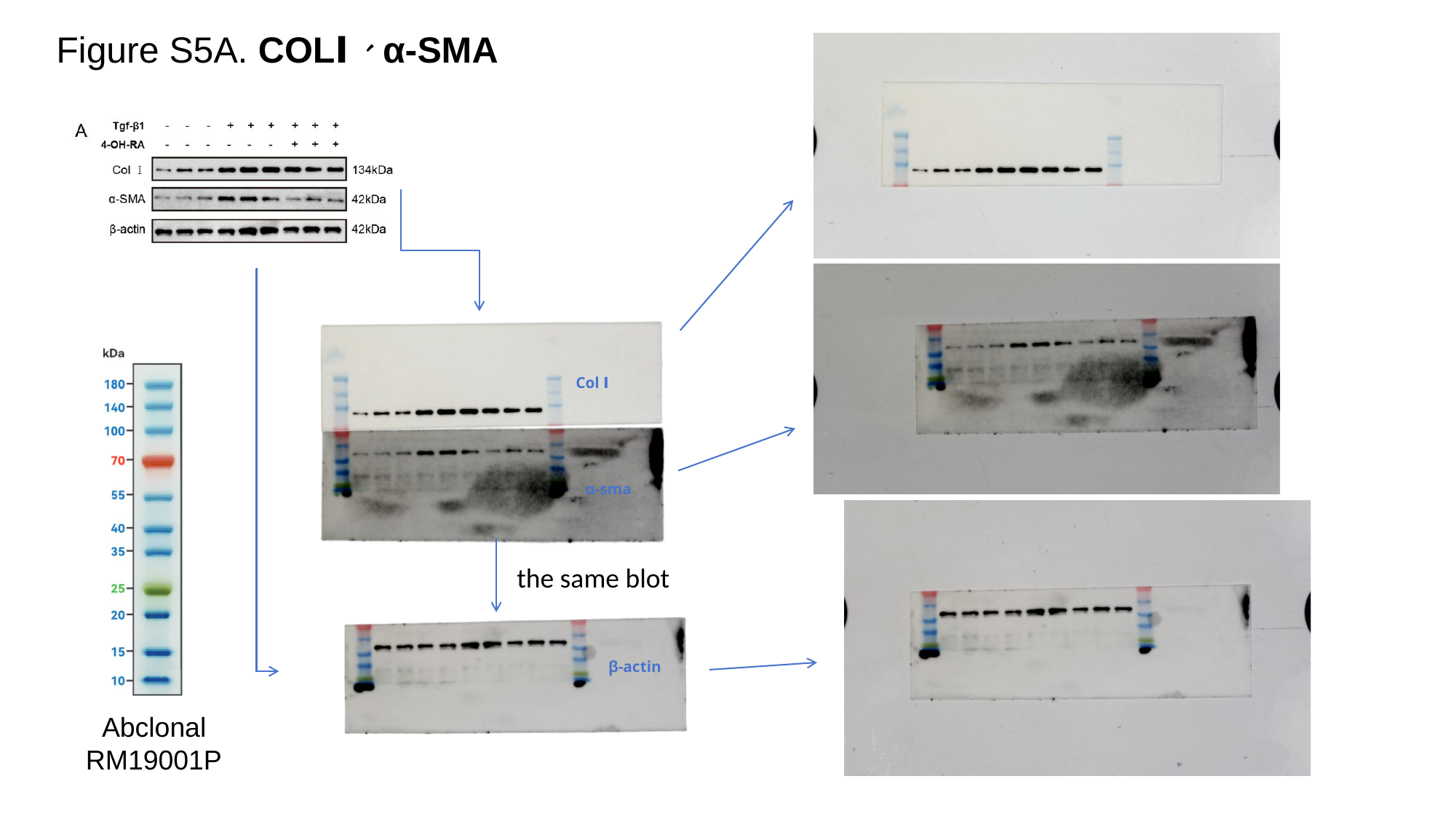

Figure S5A. COLⅠ、α-SMA
Col Ⅰ
α-sma
the same blot
β-actin
Abclonal
RM19001P

## Slide 12
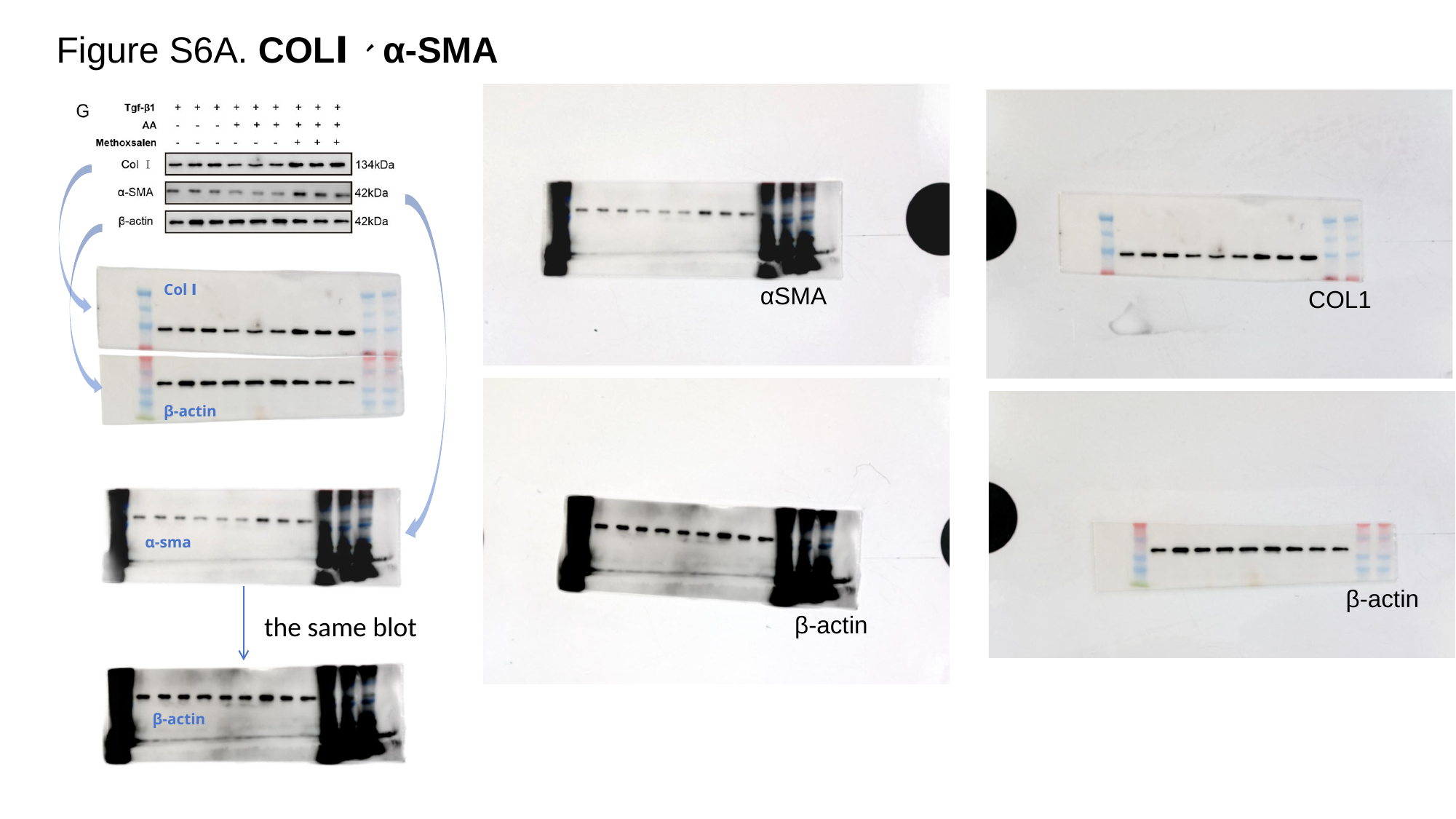

Figure S6A. COLⅠ、α-SMA
αSMA
Col Ⅰ
COL1
β-actin
α-sma
β-actin
the same blot
β-actin
β-actin

## Slide 13
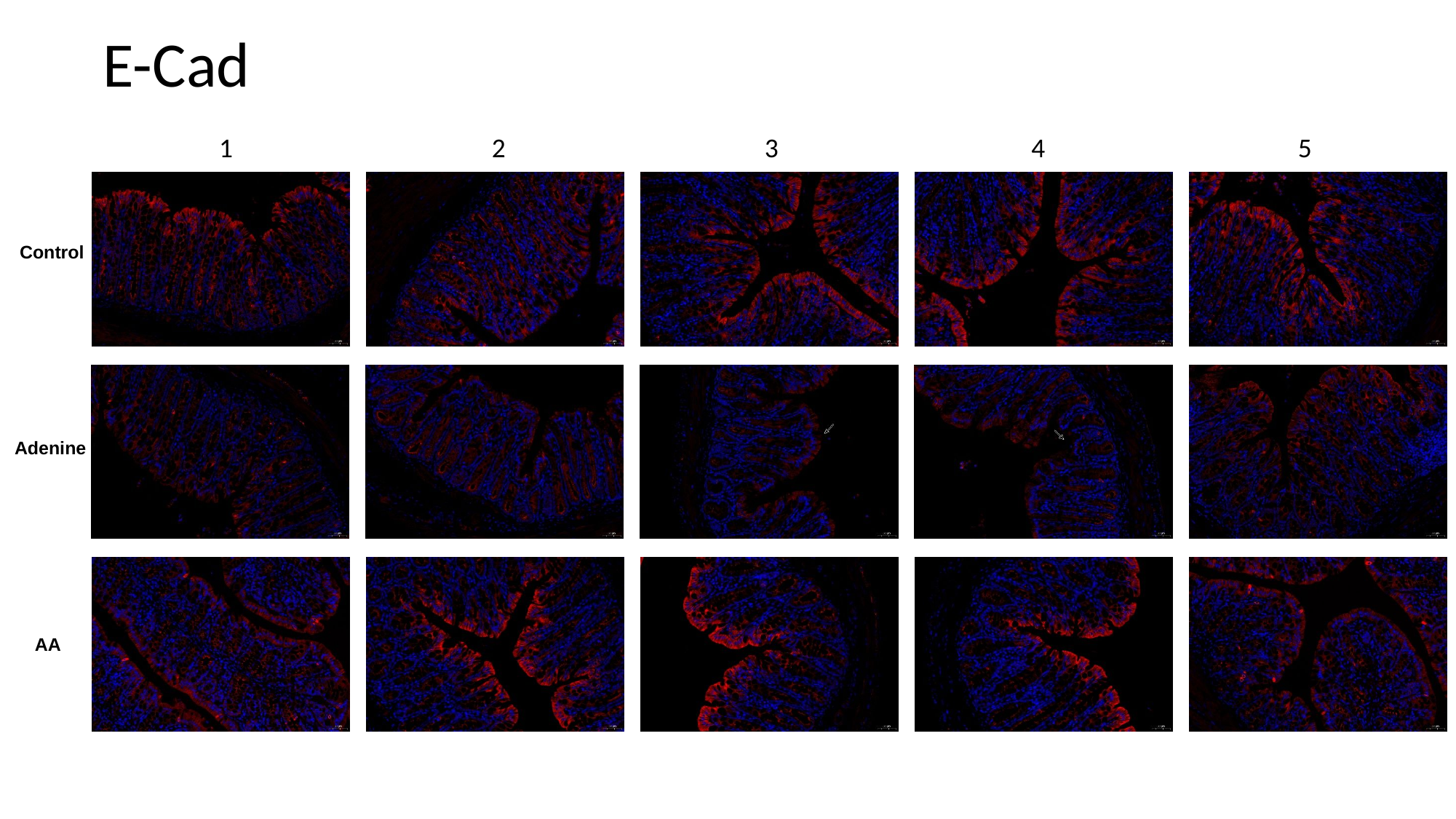

# E-Cad
1 2 3 4 5
 Control
Adenine
 AA

## Slide 14
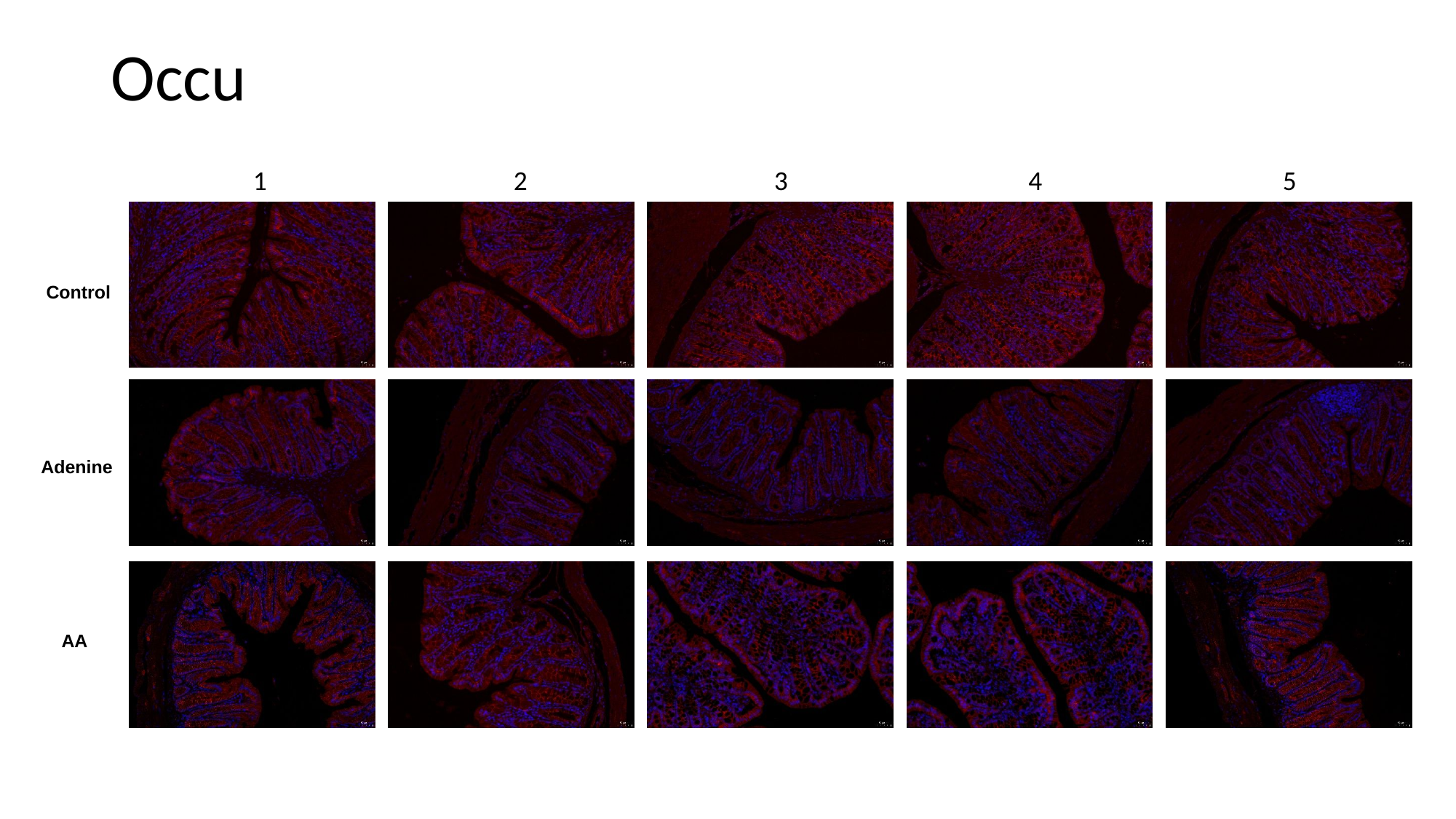

# Occu
1 2 3 4 5
 Control
Adenine
 AA

## Slide 15
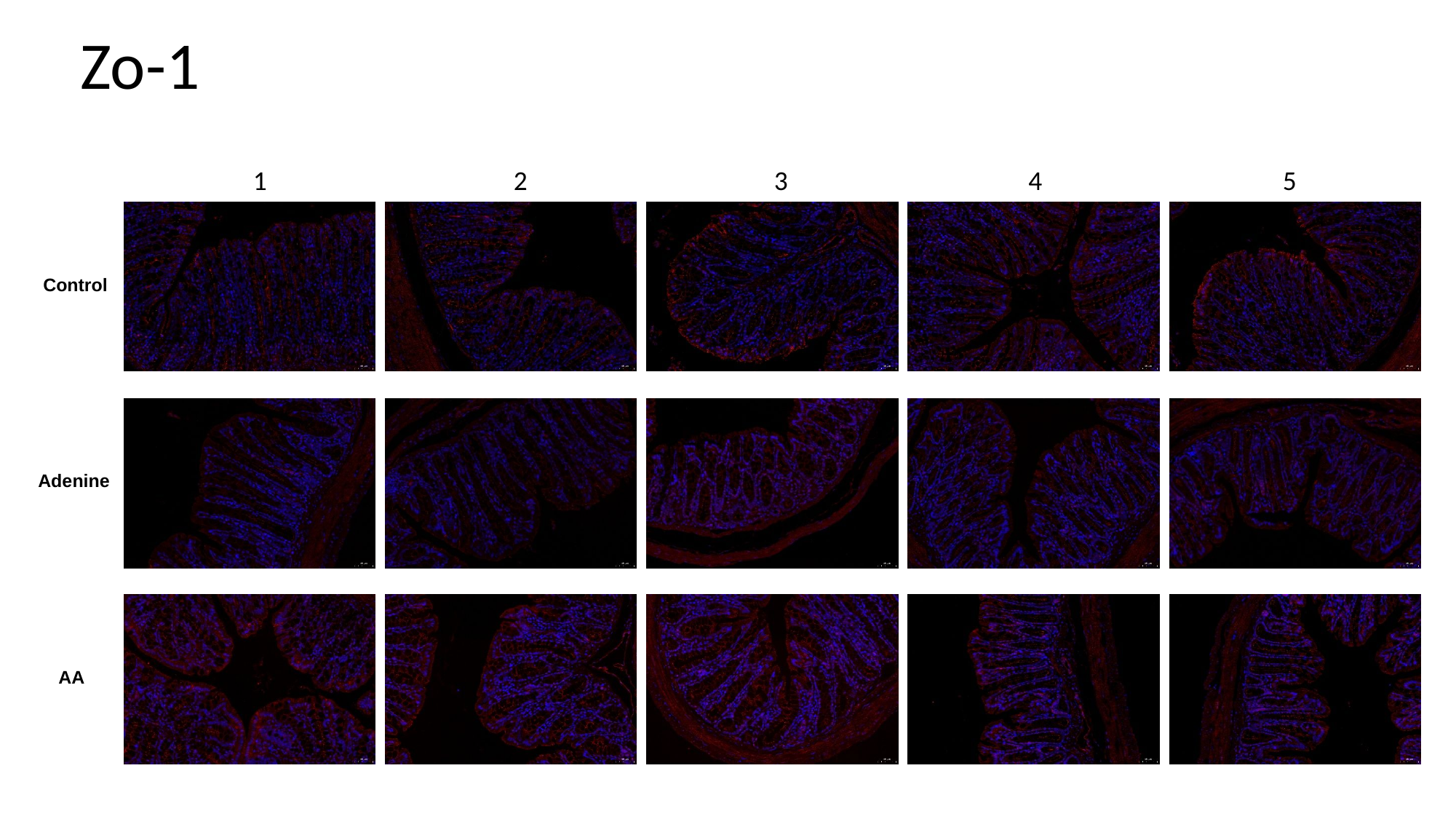

# Zo-1
1 2 3 4 5
 Control
Adenine
 AA
