# Supplementary material for: Asiatic acid from Centella asiatica alleviates renal fibrosis: coordinated modulation of the gut–kidney axis and retinol metabolism
Source: Front Nutr. 2026 Jul 10;13:1802674. doi: 10.3389/fnut.2026.1802674 (PMC13395619; doi:10.3389/fnut.2026.1802674)
Supplement: Supplementary file 2 [file Data_Sheet_1.PDF]

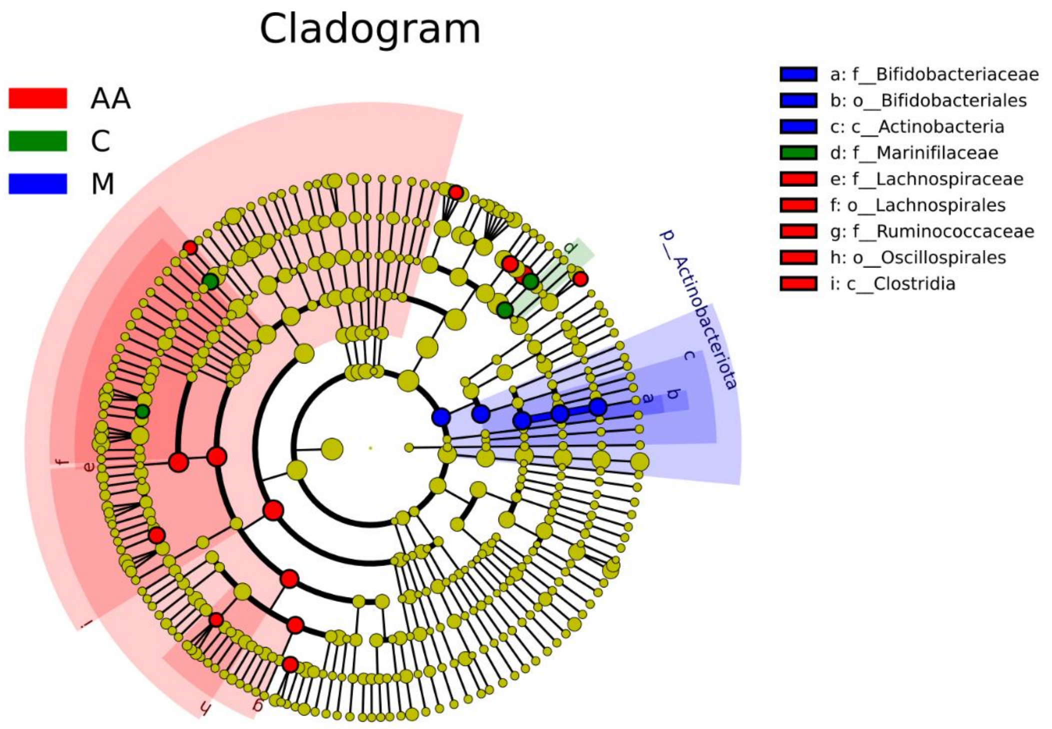

Figure 1: Taxonomic cladogram illustrating the distribution of LDA values (LDA > 2.5) for significantly different taxa across groups. C = control, M = CKD/adenine, AA = high dose AA.

Table 1: Peak intensity of detected 5 retinoids in kidneys

	Vitamin A	(9cis)-Retinal	4-Hydroxyretinoic Acid	All trans-Retinal	All-Trans-13,14-Dihydroretinol
C_1	6034315	1.6E+08	2.27E+08	2644803	873065
C_2	5659707	2.52E+08	2.78E+08	3419002	1429501
C_3	5083877	1.52E+08	2.18E+08	3644837	1191386
C_4	3315432	2.24E+08	2.16E+08	4470757	1070958
C_5	2549503	26600060	206807.5	979957.1	287272.8
M_1	776142.9	2159989	266291.4	428490.4	273931.8
M_2	717484.3	1644596	240278.7	435100.1	260992.7
M_3	750396.3	2193394	250673.1	157264.6	260286.3
M_4	755095.7	2253201	217665.4	239484.6	249455.3
M_5	1735093	1810767	223784.5	282680.7	258743.2
AA_1	4074170	31400000	72753750	2463762	683428.5
AA_2	4897489	67550798	1.8E+08	4853451	1252632
AA_3	2991077	27151670	63616638	2110710	378227.1
AA_4	3600018	50506182	65823440	2889745	633464.9
AA_5	3934584	27513595	57442056	3026849	713552.4
AA.vs.M_log2FC	2.0420809	5.595458	8.518127	3.313894	1.490068
AA.vs.M_Pvalue	0.0002773	0.00143	5.99E-06	1.76E-05	0.007097

M.vs.C_log2FC	-2.257858	-6.29777	-9.6133	-3.29638	-1.89634
M.vs.C_Pvalue	0.0001603	0.012403	0.017593	0.000214	0.013796

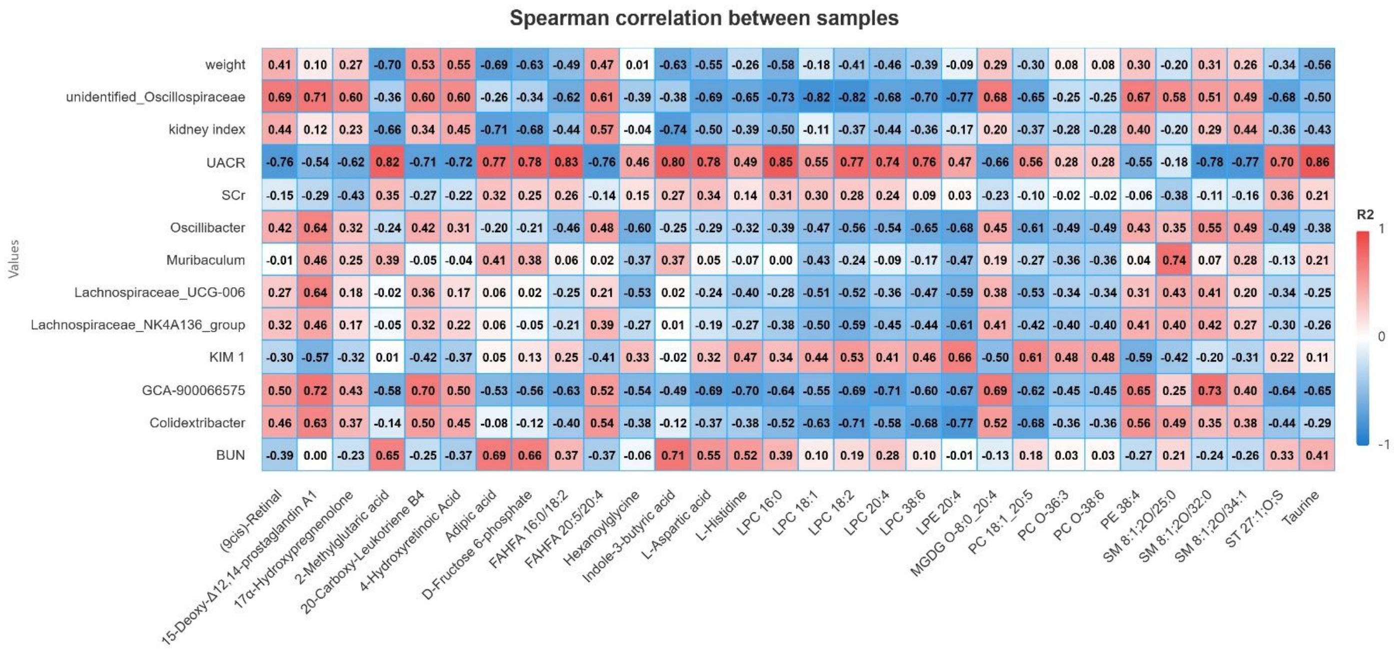

Figure 2: Heatmap of Spearman correlation analysis between critical metabolites and clinical indicators/bacterial taxa, with r-values.

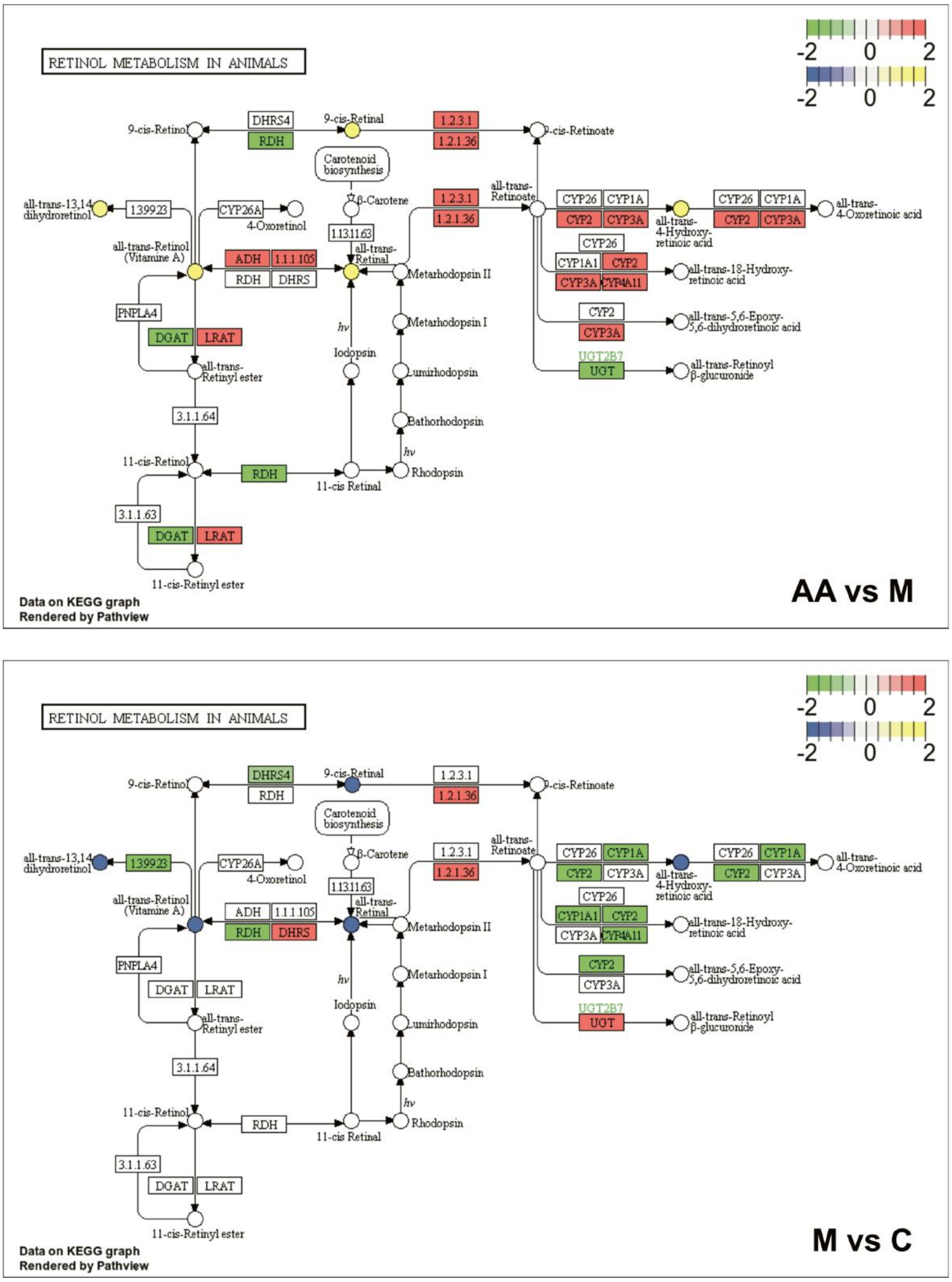

Figure 3: KEGG map of the retinol metabolism in the AA vs. M comparison and M vs. C comparison. Red borders: significantly upregulated genes; green: downregulated. Yellow borders: significantly upregulated metabolites; blue: downregulated.

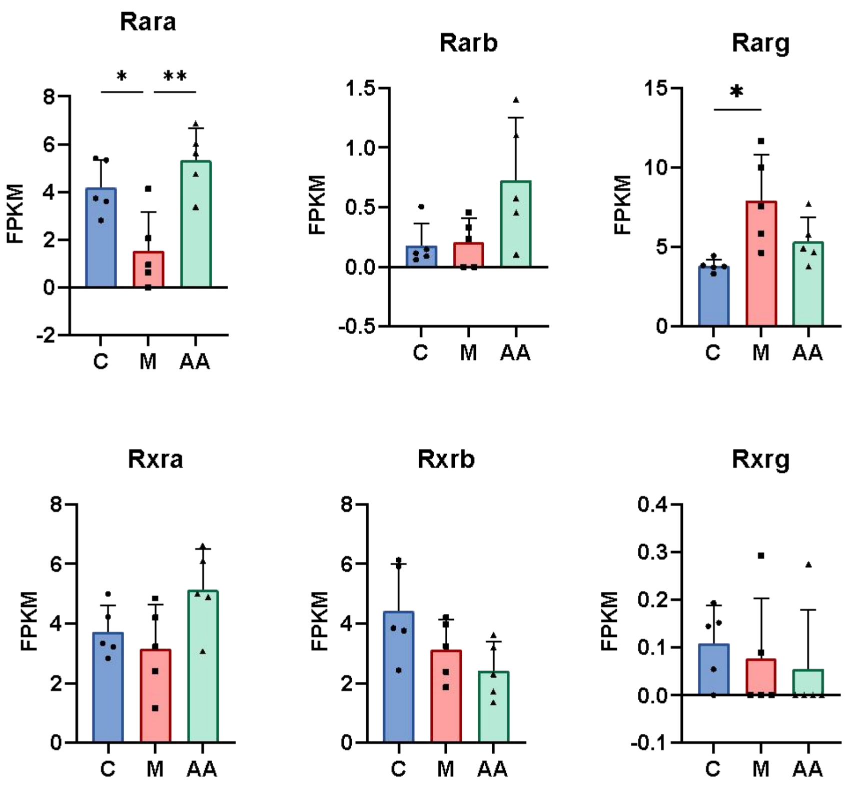

Figure 4: FPKM values for RXR isoforms (Rxra, Rxrb, Rxrg) and RAR isoforms (Rara, Rarb, Rarg) (n = 5). After normality testing, one-way ANOVA followed by Tukey's test or Kruskal-Wallis test followed by Dunn's test was used for multiple comparisons. Statistical comparisons: $*P < 0.05$, $**P < 0.01$.

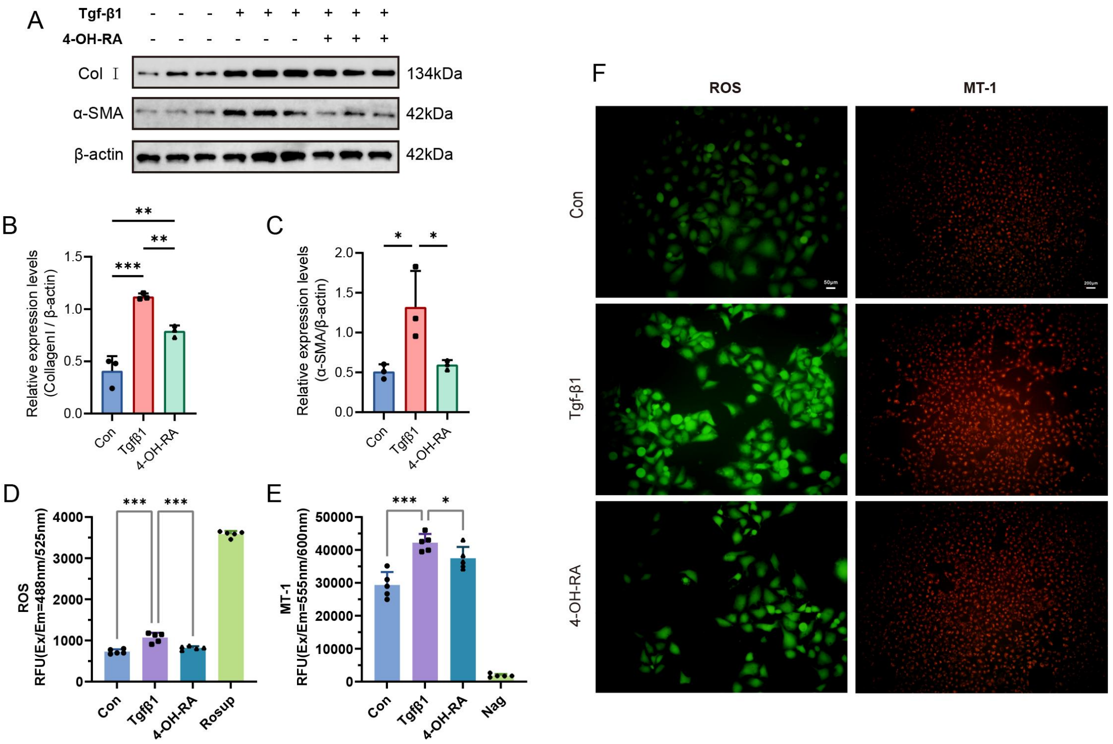

Figure 5: Western blot (A) and semi-quantification (B, C) showing the effects of 4-OH-RA (90nM) on fibrotic markers. RFU measurements of ROS (D) and MT-1 (E) in AA-treated HK-2 cells subjected to CYP inhibition. (F) Representative fluorescence images of ROS (scale bar = 50 μm)

and MT-1 (scale bar = 200 μm) staining. After normality testing, one-way ANOVA followed by Tukey's test or Kruskal-Wallis test followed by Dunn's test was used for multiple comparisons. AA = high dose AA, *P < 0.05, **P < 0.01, ***P < 0.001.

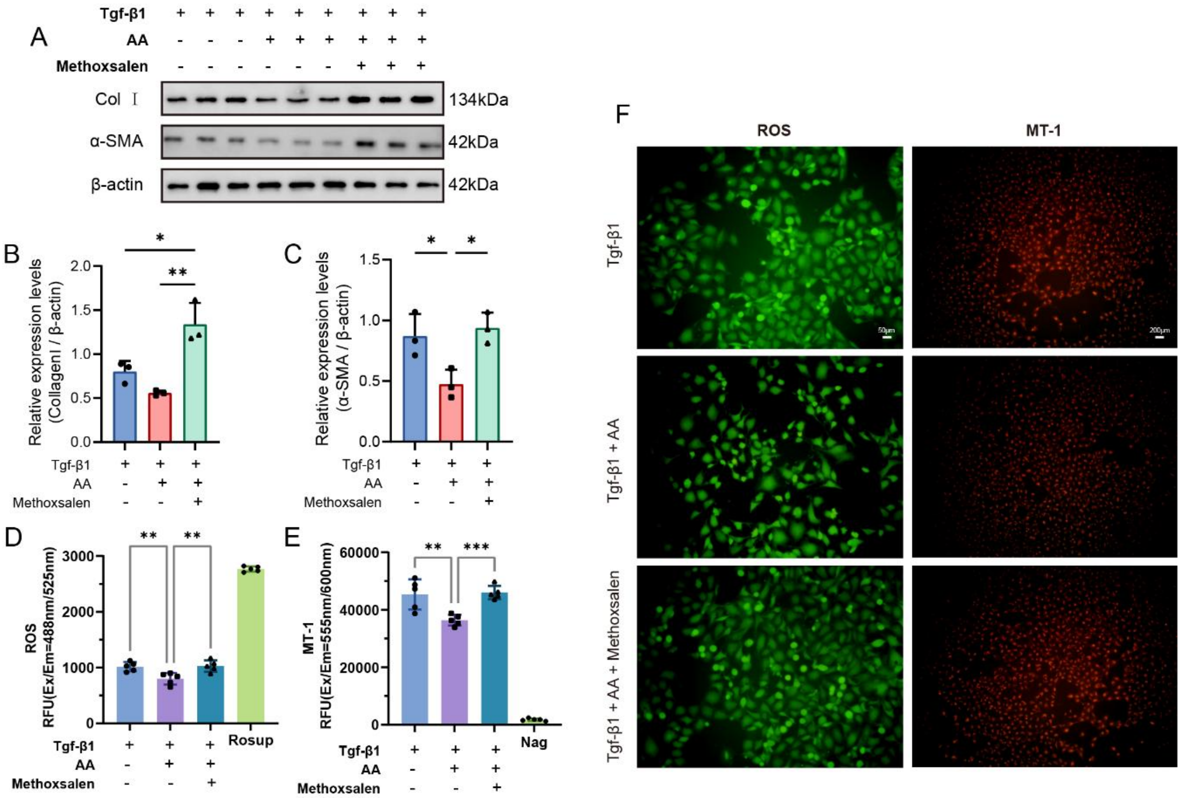

Figure 6: Western blot (A) and semi-quantification (B, C) showing the effects of AA (15μg/mL) on fibrotic markers with or without the CYP inhibitor Methoxsalen. RFU measurements of ROS (D) and MT-1 (E) in AA-treated HK-2 cells subjected to CYP inhibition. (F) Representative fluorescence images of ROS (scale bar = 50 μm) and MT-1 (scale bar = 200 μm) staining. After normality testing, one-way ANOVA followed by Tukey's test or Kruskal-Wallis test followed by Dunn's test was used for multiple comparisons. AA = high dose AA, *P < 0.05, **P < 0.01, ***P < 0.001.
